# Electrophysiological Characterization of the Venom and Toxins from the Scorpion *Tityus championi* Targeting Voltage-Gated Sodium Channels and Molecular Modeling of Tch3, a Toxin with Therapeutic Potential for Pain Relief

**DOI:** 10.3390/biom16040552

**Published:** 2026-04-08

**Authors:** Galit Akerman-Sánchez, Steve Peigneur, Kathleen Carleer, Natalia Ortiz, Felipe Navia, Leonardo Fierro, Santiago Castaño, Cecilia Díaz, Jan Tytgat, Oscar Brenes

**Affiliations:** 1 Department of Physiology, School of Medicine, University of Costa Rica, San José 11501, Costa Rica; johanna.akerman@ucr.ac.cr; 2 Postgraduate Study System, University of Costa Rica, San José 11501, Costa Rica; 3 Toxicology and Pharmacology, Department of Pharmaceutical and Pharmacological Sciences, University of Leuven (KU Leuven), 3000 Leuven, Belgium; steve.peigneur@univie.ac.at (S.P.); kathleen.carleer@kuleuven.be (K.C.); 4 Department of Biochemistry, School of Medicine, University of Costa Rica, San José 11501, Costa Rica; natalia.ortiz_c@ucr.ac.cr (N.O.); cecilia.diaz@ucr.ac.cr (C.D.); 5 Laboratory of Herpetology and Toxinology, University of the Valley, Cali 760043, Colombia; navia.johan@correounivalle.edu.co (F.N.); leonardo.fierro@correounivalle.edu.co (L.F.); santiago.castano@correounivalle.edu.co (S.C.); 6 Clodomiro Picado Institute, Faculty of Microbiology, University of Costa Rica, San José 11103, Costa Rica; 7 Neuroscience Research Center, University of Costa Rica, San José 11501, Costa Rica

**Keywords:** *Tityus*, buthidae, scorpion venom, voltage-gated sodium channels, oocytes, invertebrate neurons, *Xenopus*, *Helix*, *Cornu*, electrophysiology, molecular docking

## Abstract

Scorpion neurotoxins are small peptides that target ion channels and offer opportunities for novel therapeutic discovery. This study analyzed the functional effects of the venom and toxins from the Costa Rican endemic scorpion, *Tityus championi*. Initially, crude venom was tested on different isoforms of voltage-gated sodium channels. Our findings revealed that the venom contains toxins that affect mammalian Na_V_1.6 and Na_V_1.7, as well as the cockroach BgNa_V_1 channel. Increased currents through Na_V_1.6 and BgNa_V_1 channels were associated with bigger window currents and inhibition of inactivation. Decreased Na_V_1.7 currents were associated with smaller conductance. Crude venom and TCh3 toxin inhibited action potential generation in invertebrate neurons expressing Na_V_1.7-like channels. In these neurons, Tch2 and Tch4 toxins shifted voltage sensitivity to more negative potentials, ultimately widening the window current but decreasing channel availability. Conversely, Tch3 behaved as an inhibitory toxin, closing window currents and decreasing channel availability. Structural modeling showed that Tch3 adopts an αββ fold and binds the S3–S4 loop of Domain II in human Na_V_1.7. These data show the diverse effects of scorpion venoms on channels and neurons, characterize its principal toxins, and show that Tch3 has therapeutic potential for pain relief.

## 1. Introduction

Scorpion venom is a complex mixture of peptide neurotoxins that interfere with the normal function of the nervous system, exhibiting potent and specific activity to attack a possible offender or prey, by immobilizing or killing it [1,2]. In scorpions, evolutionary pressure has selected these peptides to modulate different ion channels [3], especially sodium (Na^+^) and potassium (K^+^) ion channels [4,5].

Voltage-gated Na^+^ channels (Na_V_s) are key players in initiating and propagating the action potentials (AP) in excitable cells, such as neurons, glands, cardiac, and skeletal muscles [6]. The AP firing in these cells is mainly dependent on Na^+^ influx through Na_V_s, and the sequential activation, inactivation, and recovery from inactivation of these channels shape the action potential waveform and the firing frequency, modulating cellular functioning [7].

Scorpion toxins selective for Na_V_s are called NaTxs, and they contain 60–76 amino acids with disulfide bridges stabilizing their tertiary structure. Based on their mechanisms of action, NaTxs have been classically classified into two types [8,9]. The α-toxins (α-NaTxs) prevent rapid channel inactivation. Functionally, this effect can prolong the duration of the AP and enhance neurotransmitter release, leading to acute pain if acting on nociceptive neurons [10,11] or flaccid paralysis due to reduced firing frequency if acting on muscles [12,13]. On the other hand, the β-toxins (β-NaTxs) promote channel opening with a negative voltage dependence shift [14]. Functionally, a shift in opening potentials to more negative values increases AP firing frequency, leading to muscle spasms, paralysis, and cardiac arrhythmias that incapacitate prey or predators [8,15,16].

Both α- and β-NaTxs can be present in the same venom. For example, *Tityus serrulatus* venom contains toxins such as Ts2, Ts4, and Ts5, classified as classic α-NaTxs for inhibiting inactivation [17,18,19]; and the toxin Ts17, classified as β-NaTx for a 5–8 mV shift in activation curves [20], all of them with different degrees of effects on different Na_V_ isoforms. Consequently, it has been proposed that scorpion venoms ultimately increase Na^+^ currents by different action mechanisms, probably causing massive neurotransmitter release [5,21], and inducing pain, paralysis, cardiac arrhythmias, and death [15].

Because of this, determining the molecular targets of scorpion venoms is crucial for understanding the mechanisms behind neuronal and systemic effects associated with envenomation [22,23]. In addition, identifying these targets opens the way for exploring potential clinical applications or channel modulators for scientific research. For example, several scorpion toxins, such as chlorotoxin, maurotoxin, and BmK AGAP, have followed this research path and now show therapeutic potential, offering promising insights into treatments for conditions such as cancer, autoimmune diseases, and neurological disorders [24,25,26,27].

Despite its importance, Costa Rican scorpion venoms are just beginning to be analyzed. In this matter, *Tityus championi* (Buthidae family), a scorpion endemic to Costa Rica and Panama [28], is a compelling subject of study due to its distinct ecological distribution and scarce knowledge of its venom mechanisms of action. Severe cases of scorpionism in Costa Rica are rare, while in Panama, there has been an increase in morbidity and fatalities, with *T. championi* being recognized as a medically significant species [29].

In 2023, Salazar et al. demonstrated that the venom and some venom fractions from *T. championi* from Panama exhibit toxicity in mammals and insects [29]. The same year, Díaz et al. published the first list of toxins present in *T. championi* venom from Costa Rica, identifying multiple putative NaTx [30]. However, the effects of *T. championi* venom, the identification of toxins, which are supposedly targeting specific channels in mammals and insects, and their mechanisms of action remain unknown. Thus, the presence of molecules with therapeutic potential has not yet been determined.

The present study aimed to characterize the electrophysiological effects of *T. championi* venom on several mammalian and insect Na_V_ isoforms expressed on *Xenopus* oocytes. Since an interesting effect was observed in Na_V_1.7, we selected an invertebrate neuronal model expressing a Na_V_1.7-like channel to characterize the venom’s effect on neuronal excitability and the electrophysiological impact of the most abundant toxins on these channels. And finally, we modeled in silico the structure of a promising toxin that inhibits invertebrate and mammalian Na_V_1.7 channels and characterized its potential interaction with human Na_V_1.7 through molecular docking.

## 2. Materials and Methods

### 2.1. Venom, Toxins Purification, and Sequencing

The venom of *T. championi* was obtained from the Laboratorio para la Investigación de Animales Peligrosos (LIAP) at the Clodomiro Picado Institute (ICP) of the University of Costa Rica. The venom collected by electrostimulation of the telson from 10 adult specimens was maintained in their animal facility, then pooled, lyophilized, and dissolved in distilled water to achieve a concentration of 7.5 mg/mL. The obtention and use of this venom was approved by the Biodiversity Commission of the University of Costa Rica (No. 293-2021).

*T. championi* toxins were purified from a pool of venoms (10 adult individuals) by a combination of size exclusion HPLC (SEC-HPLC) and reverse phase HPLC (RP-HPLC). Briefly, 2 mg of venom was dissolved in PBS, centrifuged for 5 min at 10,000× *g*, and separated by SEC-HPLC in a S2000 BioSepSEC column (10 × 300 mm) (Phenomenex, Torrance, CA, USA), equilibrated with 0.06 M NaCl, 0.02 M sodium phosphate buffer (pH 7.2), at 1 mL/min, in an Agilent 1220 chromatograph (Agilent Technologies, Inc., Santa Clara, CA, USA), monitoring at 215 nm. The two most prominent fractions, which elute at 9.845 and 11.030 min, were collected and dried in a SpeedVac (Termo Fisher Scientific, Waltham, MA, USA). These fractions were dissolved in distilled water and further separated by RP-HPLC on a C18 column (250 × 4.6 mm, 5 µm particle size; Phenomenex, Torrance, CA, USA) using an Agilent 1220 chromatograph with monitoring at 230 nm. Elution was performed at 1 mL/min by applying a linear gradient of acetonitrile containing 0.1% TFA for 60 min. Tch2 and Tch3 elute at the 11.030-min fraction, whereas Tch4 elutes at the 9.845-min fraction. Peptide sequencing was performed by mass spectrometry according to Díaz et al. (2023) [30].

### 2.2. Xenopus laevis Oocyte Isolation and Heterologous Expression

All experiments with oocytes received approval from the Ethical Committee for Animal Research at KU Leuven (code 074/2023). Procedures involving *Xenopus* heterologous expression were performed as previously described [31]. Briefly, stage V-VI oocytes were surgically extracted from young *X. laevis* frogs anesthetized with 0.1% buffered tricaine (ethyl 3-aminobenzoate methanesulfonate, 1 g/L, Sigma-Aldrich, Rockville, MD, USA) and NaHCO_3_ (sodium bicarbonate, 1 g/L; Sigma-Aldrich, USA) in aquarium water (pH 7.5). Following the recovery period, the frogs were monitored daily and returned to their tanks at the Aquatic Facility of KU Leuven. The ovarian lobes underwent enzymatic defolliculation in a Ca^2+^-free ND96 solution (96 mM NaCl, 2 mM KCl, 2 mM MgCl_2_, and 5 mM HEPES), supplemented with collagenase from *Clostridium histolyticum* type IA, 1.5 mg/mL; Sigma-Aldrich, USA, for 2.5 h at 16 °C. Single oocytes were transferred to a calcium-containing ND96 buffer (96 mM NaCl, 2 mM MgCl_2_, 2 mM KCl, 5 mM HEPES, and 1.8 mM CaCl_2_; pH 7.5) supplemented with gentamicin (100 mg/L; Schering-Plough, Heist-op-den-Berg, Belgium) and theophylline (90 mg/L; ABC chemicals, Nazareth, Belgium) at 16 °C.

Selected oocytes were injected with 5–20 nL of cRNA at a concentration of 1 ng/nL using a micro-injector (Micro2T SmarTouchTM, World Precision Instruments, Sarasota, FL, USA). Electrophysiological experiments were conducted following cRNA injection, with an incubation period of 1–5 days at 16 °C in ND96 buffer. The cRNA used encoded rNa_V_1.2, rNa_V_1.4, hNa_V_1.5, rNa_V_1.6, rNa_V_1.7, and BgNa_V_1 and their auxiliary subunits rβ1, hβ1, and TiPE β.

### 2.3. Electrophysiological Recordings and Treatment in Xenopus Oocytes

Two-electrode voltage-clamp (TEVC) recordings were performed with a GeneClamp 500 amplifier (Molecular Devices, Downingtown, PA, USA), under the control of pClamp data acquisition system, and pClamp Clampex 10.4 software (Molecular Devices, San Jose, CA, USA). Currents were recorded at room temperature (18–22 °C) in a 200 µL recording chamber, before and after venom treatment. All channels were tested at two venom concentrations (0.075 µg/µL and 0.150 µg/µL), and purified Tch3 in rNa_V_1.7 was tested at 0.006 µg/µL.

For recordings, borosilicate glass electrodes were pulled using a microelectrode puller, PUL-1 (World Precision Instruments, Sarasota, FL, USA), and filled with 3 M KCl (resistance: between 0.5 and 1.5 MΩ). Recorded currents were sampled at 20 kHz and filtered through a four-pole low-pass Bessel filter at 2 kHz. Leak subtraction was performed using a −P/4 protocol. During recordings, leak currents were monitored, and the cells with increased leakage and loss of reversal potential were discarded.

Venom effect was monitored through 100 ms depolarizations to 0 mV from a baseline of −90 mV. Standard current-voltage (I-V) relationships were performed using 50 ms step depolarizations ranging from −80 to +65 mV in 5 mV increments from a −90 mV baseline. For steady-state inactivation analysis, a two-step protocol was used involving a 100 ms prepulse from −80 to 0 mV with 5 mV steps, followed by a test pulse to 0 mV. The interstimulus interval of the protocols was 15–50 ms.

To obtain the channel opening probability, the conductance (*g_Na_*) was calculated using Equation (1), where *I_peak_* is the peak current recorded, *V_m_* is the voltage step, and *V_rev_* is the reversal potential.

(1) $$g_{Na}=\frac{I_{peak}}{\left(V_{m}-V_{rev}\right)}$$

The apparent opening probability (Po) of m and h particles was calculated by normalizing the conductance with the maximum conductance or the maximal current, respectively, and plotted as a function of voltage. The data were fitted using modified Boltzmann equations for steady-state activation (02) and steady-state inactivation (03), where *V*_1/2_ represents the voltage at half-maximal activation or half-maximal inactivation, and *k* is the slope factor for voltage dependence.

(2) $$I\left(V\right)=\frac{Gmax}{1+e^{-\left(\frac{V-V1/2}{k}\right)}}$$

(3) $$I\left(V\right)=\frac{Imax}{1+e^{\left(\frac{V-V1/2}{k}\right)}}$$

For maximum conductance (*G_max_*) determination, the I-V relationship of the Na_V_ channel was fitted using the Boltzmann Equation (4), where *V_rev_* is the reversal potential, *z_act_* is the apparent gating valence, *F* is the Faraday constant, *R* is the gas constant, and *T* is the temperature.

(4) $$I\left(V\right)=\frac{G_{max}(V-V_{rev})}{1+{exp}^{-z_{act}(V{-V}_{1/2})F/RT}}$$

Steady-state activation and steady-state inactivation were plotted as a function of voltage, and the area resulting from the overlap of both sigmoidal curves was defined as the window current.

For some channels, the fast and slow components of the current decay time constant (tau) were calculated at the voltage of maximum venom effect, fitting the current with a standard two-component exponential equation.

### 2.4. C1 Helix Neuron Isolation

Juvenile specimens of the terrestrial snail *Helix aspersa* (NCBI taxonomic ID: 6535, synonym *Cornu aspersum*) were provided by local breeders. The snails were housed in plastic boxes in a room with a regulated temperature (20 °C) and a 12:12 h light:dark cycle. Snails were fed once a week with a calcium-rich diet and lettuce or cucumber. Before procedures, a 0.1 M MgCl_2_ solution was injected into the snail’s foot to anesthetize and induce muscle relaxation. Subsequently, they were sacrificed by evisceration. All methodologies were previously validated by the Institutional Animal Care and Use Committee (CICUA-075-2019) of the University of Costa Rica, and efforts were made to minimize animal numbers and suffering in accordance with the guidelines established by the Ethical-Scientific Committee on the protection of animals for scientific purposes.

Cell isolation was performed as previously described [32]. Briefly, isolated cerebral ganglia were incubated for enzymatic digestion using protease type XIV (Sigma-Aldrich, St. Louis, MO, USA) in L15 isotonic medium (0.4 U/mL) at 34 °C for 4 h. Following digestion, the ganglia were washed with L15 medium to isolate C1 neurons via microsurgery. This neuron was identified by its position, size, and distinctive morphology in the cerebral ganglion. Neurons were gently isolated with segments of the axon still attached to the soma and subsequently placed in dishes pre-coated with 5% bovine serum albumin (BSA) until the axon was reabsorbed, resulting in a phenotype known as the soma-configuration, as previously described [33].

### 2.5. Electrophysiological Recordings and Treatment in Helix Neurons

Standard patch-clamp recording techniques, in current- and voltage-clamp configurations, were used with isolated cells under a Nikon inverted microscope (Eclipse TS100). Signals were amplified using a Multiclamp 700B amplifier, under the control of pClamp 11 data acquisition system (Molecular Devices, San Jose, CA, USA). Currents and voltages were recorded at room temperature in a 2 mL recording chamber, before and after venom and toxin treatments. During experiments, venom and toxins were added directly to the culture dish and tested at a concentration of 0.01 µg/µL.

For recordings, borosilicate glass electrodes were pulled using a Flaming/Brown P-1000 microelectrode puller (World Precision Instruments, Sarasota, FL, USA). For Na^+^ current recording, the electrodes were filled with an intracellular solution consisting of (mM): 110 CsCl, 10 HEPES, 2 MgCl_2_, 1 NaCl, 2 EGTA, pH 7.4 (with CsOH); resistance between 1.5 and 3.0 MΩ. The extracellular solution was (mM): 4 KCl, 90 NaCl, 1 CaCl_2_, 5 MgCl_2_, 10 Tris, 30 TEA-HCl, 4 4-AP, pH 7.4 (with NaOH). All solutions were adjusted to a pH of 7.4 before use. For action potential recordings, the electrodes were filled with an intracellular solution consisting of (mM): 3 NaCl, 100 KCl, 1 MgCl_2_, 5 EGTA, 10 HEPES, pH 7.4 (with KOH). The extracellular solution consisted of the L15 medium modified for *Helix* neurons and contained (mM): 6.97 CaCl_2_, 4.92 MgCl_2_, 0.35 MgSO_4_, 5.34 KCl, 0.19 KH_2_PO_4_, 66.52 NaCl, 0.574 Na_2_HPO_4_, pH 7.6 (with NaOH).

The voltage-clamp was used to measure Na^+^ currents. Data were sampled at 20 kHz, and series resistance compensation was set to 60–80% before data collection, depending on the cell. During recordings, access resistance, membrane resistance, and cell capacitance were monitored, and cells that lost resistance, reversal potential, or resting membrane potential were discarded.

To evaluate Na_V_ channel activation, currents were measured at 200 ms steps from −60 mV to +60 mV, with 10 mV increments, from a baseline of −50 mV. For the steady-state inactivation, a similar prepulse was used, followed by a 200 ms test pulse at −20 mV. The interstimulus interval of both protocols was 230–500 ms. For voltage-dependent activation, the conductance (gNa), channel opening probability (Po), and steady-state inactivation were fitted using Equations (1)–(4). The area subtended by the intersection of the activation and inactivation curves (called window current) was calculated with RStudio (Version 2023.06.0+421). The Calculation Code is available at https://github.com/GalitAkerman/Statistics-Helix-Article (accessed on 4 April 2026). The time required for the channels to recover from the inactive state (recovery from inactivation) was measured using two voltage steps at −20 mV, separated by an increasing interpulse interval (5–80 ms) with a Δt = 5 ms.

The stimulation protocol for current-clamp recordings to measure firing frequency consisted of three stimuli of increasing intensity (0.5, 1.0, and 1.5 nA), each lasting 500 ms and with an interstimulus interval of 3 s. The Mean Firing Frequency (MFF) was reported in Hz.

### 2.6. Protein Modeling of Tch3 Toxin

After testing three different toxins (Tch2, Tch3, and Tch4), one of them showed interesting effects on invertebrate and mammalian Na_V_1.7 channels. We decided to model the Tch3 toxin and its possible interaction with the human Na_V_1.7 channel. The three-dimensional structure of the Tch3 toxin (sequence: EALDGYPLSKNNYCKIYCPNDEVCKDTCKHRAGATNGKGDCIWQTC YCYDVAPGTK) was predicted using AlphaFold2 implemented in Google ColabFold (v1.6.1) [34]. Six recycling iterations and 200 relaxation steps were performed to ensure model convergence and structural consistency. The final predicted model was relaxed using the Amber force field to optimize local stereochemistry and relieve steric clashes. The resulting structure was visually inspected and validated before docking experiments.

### 2.7. Human Na_V_1.7 Preparation and Protein-Protein Docking Protocol

#### 2.7.1. hNaV1.7 Structural Refinement and Membrane Interaction

The human Na_V_1.7 (Protein Data Bank ID: 7W9K; Cryo-EM structure of the human Na_V_1.7–β1–β2 complex at 2.2 Å resolution) was selected as the receptor model. Since some extracellular loops were partially unresolved in the experimental structure, AlphaFold was employed to reconstruct and refine the missing regions, yielding a complete topology of the extracellular domain. This step was essential because previous studies have shown that peptide toxins primarily interact with these extracellular regions [35,36].

For the membrane system construction, the refined channel structure was embedded into a lipid bilayer using CHARMM-GUI, generating a membrane system composed of 80% POPC and 20% cholesterol [37]. This lipid composition was selected to approximate the neuronal membrane environment and preserve the steric and dielectric properties characteristic of the physiological context.

For energy minimization and equilibration, the protein–membrane system was equilibrated using GROMACS version 2024.3. The system underwent an initial energy minimization using the steepest descent algorithm, followed by NPT ensemble equilibration to stabilize pressure and temperature. Two additional equilibration phases were conducted to ensure full relaxation of the lipid and solvent environment, after which a final minimization was performed.

#### 2.7.2. Docking Configuration, Replicates, and Result Selection

Protein–protein docking simulations were performed using the HDOCK server (http://hdock.phys.hust.edu.cn/, accessed on 1 October 2025). The Na_V_1.7 channel, including its membrane-embedded form, was defined as the receptor, while the Tch3 toxin served as the ligand. To restrict the docking search to biologically relevant regions, the following extracellular residues of Na_V_1.7 were defined as potential binding sites: Domain I S3–S4 (247–256), Domain II S3–S4 (866–875), Domain III S3–S4 (1321–1329), Domain IV S3–S4 (1642–1651), and turret regions (347–355, 942–947, 1395–1401, 1742–1744). These regions correspond to the extracellular loops of each domain and the pore turret, which are known to participate in toxin binding [38].

The docking scores provided by HDOCK are reported in arbitrary units (a.u.), representing a dimensionless empirical measure of binding affinity derived from a hybrid scoring function that integrates shape complementarity, desolvation, and knowledge-based energy terms. These values are not equivalent to physical binding free energies (kcal/mol). Still, they can be used to compare relative affinities among different binding sites or docking poses within the same system. More negative scores indicate stronger predicted binding affinities.

Each docking experiment was performed in triplicate to account for stochastic variation in the search algorithm. The 10 highest-ranked binding models from each replicate (based on docking scores) were extracted, yielding a total of 30 binding energy values for subsequent statistical analysis.

#### 2.7.3. Structural and Energetic Characterization of Complexes

To characterize interaction interfaces, the top-ranked docking models were analyzed using UCSF Chimera (version chimera-1.19). Each protein–protein complex (Na_V_1.7–Tch3) was prepared by adding polar hydrogens to both receptor and ligand using the AddH tool in Chimera (University of California, San Francisco, CA, USA), assuming a physiological pH of 7.4. The explicit inclusion of polar hydrogens is essential for accurately representing intermolecular interactions, as these atoms directly participate in hydrogen bond formation and influence electrostatic orientation between molecules. Their presence enables more precise identification of hydrogen bond donors and acceptors, thereby improving the interpretation of specific contacts at the protein–protein interface.

Hydrogen bonds, salt bridges, and hydrophobic contacts were identified and quantified for each model. Residue contacts were considered significant when interatomic distances were ≤4.0 Å, including both polar and nonpolar atoms at the interface. This analysis provided an integrated description of the electrostatic, polar, and dispersive contributions stabilizing Tch3 toxin binding to hNa_V_1.7. It served as the basis for subsequent dynamic and energetic evaluations of the complex.

### 2.8. Statistical Analysis

For the functional analysis, all data were presented as means ± standard error of the mean (s.e.m.). Graphical representations were created with GraphPad Prism version 10 (GraphPad Software, Boston, MA, USA). Statistical analyses were performed using R version 2026.01.0+392, GraphPad Prism version 10, and PAST version 4.03.

Given the non-normal distribution and heteroscedasticity of the electrophysiological data, Generalized Additive Models were employed with a Gaussian location-scale family for current data, while activation and inactivation variables were modeled with standard Gaussian distributions. All models included treatment as a fixed effect, treatment-specific voltage smooth terms, and cell-level random intercepts to account for repeated measures. Model adequacy was assessed through residual diagnostics, including Q-Q plots and homoscedasticity checks. For voltage-level comparisons between treatments, model-based estimated marginal means were extracted with pairwise contrasts at each voltage step. Comparison corrections were applied using both Bonferroni and Benjamini–Hochberg false discovery rate procedures. The significance level was set at *p* < 0.05. The complete code for all statistical analyses, the values obtained, and *n* evaluated are available at (https://github.com/GalitAkerman/CR-ScorpionVenom-Nav.git, accessed on 4 April 2026).

For paired comparisons, normal distribution was assessed, and Student’s *t*-test or the Wilcoxon test was used when appropriate. For the structural analysis, to assess reproducibility and clustering among docking solutions, binding energy scores obtained from HDOCK were analyzed using Principal Component Analysis (PCA) and one-way ANOVA, followed by Tukey’s HSD test. For each docking replicate, the most negative binding energy value (corresponding to the highest predicted affinity) was selected from three independent runs. These selected energies were used to evaluate the distribution of docking conformations and to assess statistically significant differences among interaction sites. PCA was applied to explore clustering patterns and variance distributions, while boxplots were generated to visually compare binding affinities across docking sites.

## 3. Results

### 3.1. Venom Effect on Na_V_ Peak Currents

To perform a screening of *T. championi* venom effects, the mammalian isoforms Na_V_1.2, Na_V_1.4, Na_V_1.5, Na_V_1.6, and Na_V_1.7, and the insect BgNa_V_ channel were expressed in *Xenopus laevis* oocytes, and the venom was tested at two concentrations (0.075 µg/µL and 0.150 µg/µL). In the mammalian channels, both excitatory and inhibitory effects were observed.

**Figure 1:**
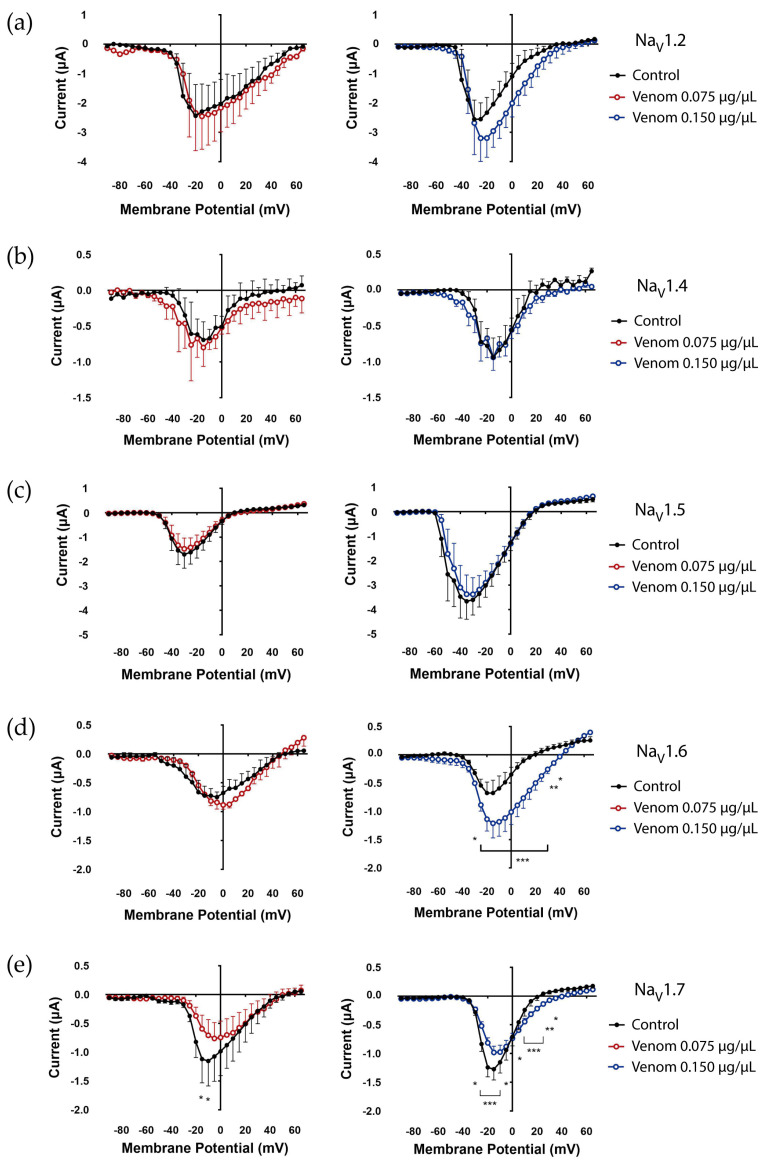
Effect of *T. championi* venom on mammalian isoforms of Na_V_ channels. Control currents were measured before (black closed circles) and after venom exposure, 0.075 µg/µL (red open circles) and 0.150 µg/µL (blue open circles). The following channel isoforms were tested: (**a**) Na_V_1.2, (**b**) Na_V_1.4, (**c**) Na_V_1.5, (**d**) Na_V_1.6, and (**e**) Na_V_1.7. Each value indicates mean ± s.e.m. The number of replicates (*n*) was as follows (0.075 and 0.150 µg/µL, respectively): Na_V_1.2 (2, 3), Na_V_1.4 (3, 4), Na_V_1.5 (2, 8), Na_V_1.6 (2, 3), Na_V_1.7 (4, 6). * = *p* < 0.05, ** = *p* < 0.01, *** = *p* < 0.001.

**Figure 2:**
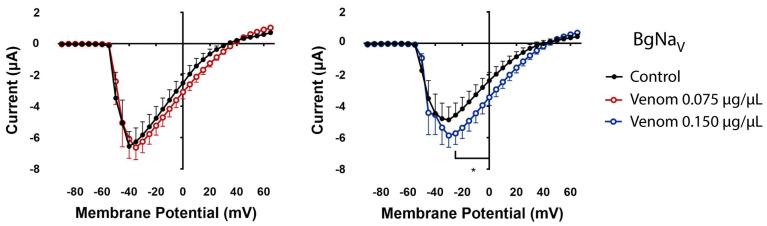
Increased current in the cockroach Na_V_ channel by *T. championi* venom. Control currents were measured before (black closed circles) and after venom exposure, 0.075 µg/µL (red open circles) and 0.150 µg/µL (blue open circles). Each value indicates mean ± s.e.m. The number of replicates (*n*) was 8 and 9 (for 0.075 and 0.150 µg/µL, respectively). * = *p* < 0.05.

For excitatory effects, the Na_V_1.2 exhibited a non-significant increase in peak currents upon exposure to venom (0.150 µg/µL) compared to the control conditions (Figure 1a). Na_V_1.6 currents increased after venom treatment, especially at 0.150 µg/µL (Figure 1d). Similarly, the cockroach BgNa_V_1 channel, an invertebrate Na_V_ channel, showed an increase in peak currents upon exposure to 0.150 µg/µL venom (Figure 2).

Towards inhibition, an effect on Na_V_1.7 currents was observed. At both venom concentrations (0.075 and 0.150 µg/µL), a decrease in the peak currents was evident at negative potentials. In contrast, at positive voltages, an increase in peak currents was observed at 0.150 µg/µL (Figure 1e).

All other vertebrate Na_V_ channels tested showed no significant differences or trends in peak currents following exposure to *T. championi* venom (Na_V_1.4, Figure 1b; Na_V_1.5, Figure 1c). The reversal potentials under control conditions and after venom exposure for Na_V_1.2, 1.4, 1.6, and 1.7 were compared; no significant changes were observed.

### 3.2. Window Currents, Inactivation Kinetics, and G_max_ of Affected Na_V_ Channels

To determine whether the changes observed in I-V relationships were due to modifications in window current, the relative conductance of channels showing noticeable trends or significant effects was plotted as a function of the applied voltage.

**Figure 3:**
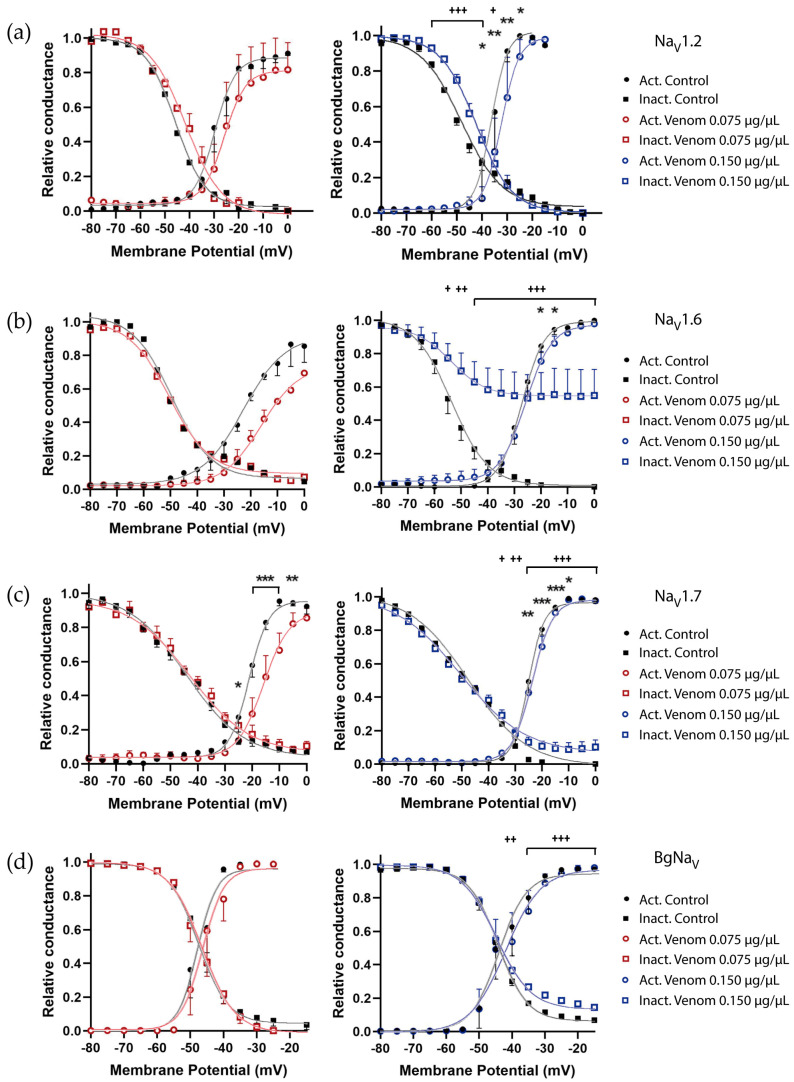
The venom alters the window current in different ways depending on the target Na_V_ expressed and the venom concentration. The window current is observed by combining the activation curves (circles), calculated as relative conductance (*G*/*G_max_*), and the inactivation curves (squares), calculated as relative current (*I*/*I_max_*), and plotting them against the voltage tested. The control open probabilities are reported before (black closed), and after venom exposure (0.075 µg/µL red open, and 0.150 µg/µL blue open). Lines represent the Boltzmann equation fitting of the data. The following channel isoforms were tested: (**a**) Na_V_1.2, (**b**) Na_V_1.6, (**c**) Na_V_1.7, and (**d**) BgNa_V_. Each value indicates mean ± s.e.m. The number of replicates (*n*) was as follows (0.075 and 0.150 µg/µL, respectively): Na_V_1.2 (2, 3), Na_V_1.6 (2, 3), Na_V_1.7 (4, 6), BgNa_V_ (8, 8). For activation comparison * = *p* < 0.05, ** = *p* < 0.01, *** = *p* < 0.001; for inactivation comparison + = *p* < 0.05, ++ = *p* < 0.01, +++ = *p* < 0.001.

For Na_V_1.2, both curves displayed consistent small rightward voltage shifts (*V*_1/2_ about +4 mV in activation and +5 mV in inactivation curves) (Figure 3a), suggesting an altered voltage sensitivity (Supplementary Tables S1 and S2). The maximum conductance (*G_max_*) was also calculated using the Boltzmann equations. A small, not significant increase from 0.057 ± 0.004 mS before venom exposure to 0.065 ± 0.009 mS after 0.150 µg/µL venom exposure was observed, which correlates with the I-V relationship trend.

For Na_V_1.6, one dominant effect was observed. At 0.150 µg/µL, a partial inactivation was evident, where the curve scarcely reached 50% relative conductance, resulting in a rightward shift of +26.3 mV in the inactivation *V*_1/2_ and a notable increase in the voltage range where the channel can be active (Figure 3b, right panel, and Supplementary Table S2), which could correlate with the increased current observed in the I-V relationship. On the other hand, under 0.075 µg/µL venom treatment, the activation curve showed a trend suggesting a smaller voltage sensitivity in the steady-state activation with a positive shift in *V*_1/2_ compared to the control condition (Figure 3b left panel, and Supplementary Table S1).

**Figure 4:**
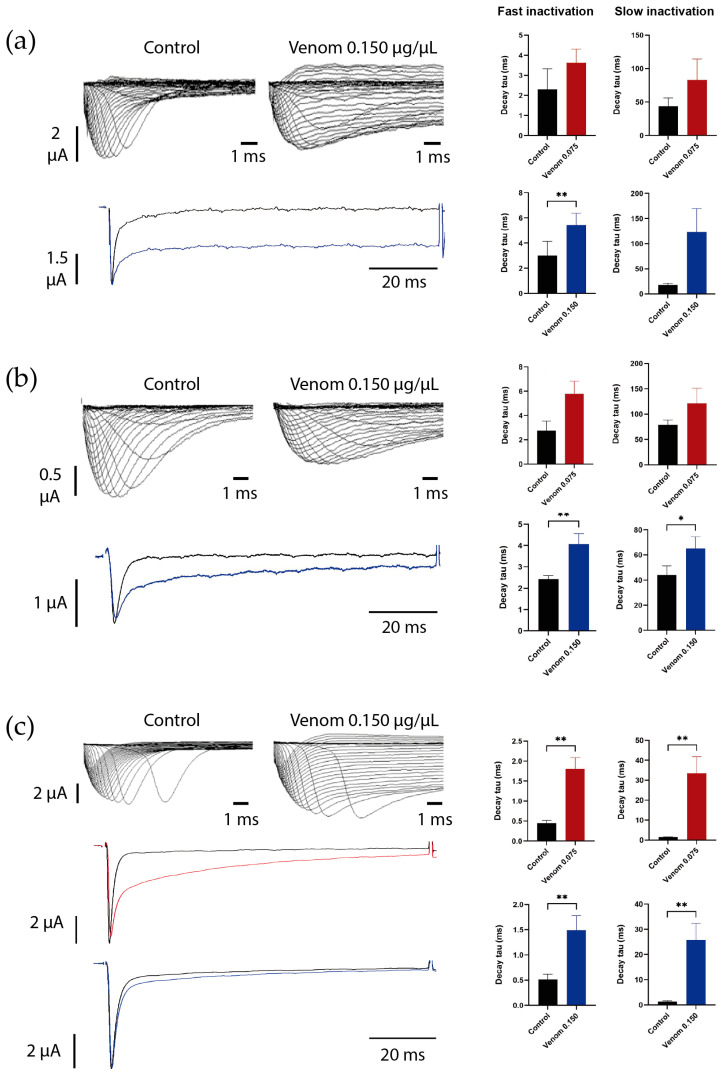
Inhibition of inactivation by venom in affected Na_V_ channels: (**a**) Na_V_1.6, (**b**) Na_V_1.7, and (**c**) BgNa_V_1. Left panels show representative current recordings evoked by a voltage-step protocol ranging from −90 to +65 mV with a 5 mV increment, with one recording extracted for comparison between control (black) and venom treatment at 0.075 µg/µL (red) or 0.150 µg/µL (blue). The right panels show the time constant of fast and slow inactivation (decay tau). Each value indicates mean ± s.e.m. The number of replicates (*n*) was as follows (0.075 and 0.150 µg/µL, respectively): Na_V_1.6 (2, 4), Na_V_1.7 (5, 8), and BgNa_V_1 (8, 9). ns = no significant, * = *p* < 0.05, ** = *p* < 0.01.

In addition, during recordings, we observed that the venom partially inhibited the inactivation of Na_V_1.6 currents, as illustrated in Figure 4a (left panel). Both fast and slow decay tau were slowed, showing a trend toward longer time constants after 0.150 µg/µL exposure, leading to a residual current. However, this effect reached statistical significance only in the fast decay tau (t_(3)_ = 6.39, *p* = 0.007) (Figure 4a, right panel), likely due to the greater variability, as reflected by the higher deviation, on slow decay tau. Both increased window current and partial inhibition of inactivation can evoke the increase in currents observed in the I-V relationship.

For the Na_V_1.7 channel, a rightward shift was observed in activation curves at both venom concentrations. Especially, under 0.075 µg/µL venom treatment, the *V*_1/2_ shifted around +6 mV, and the slope factor (*k*) increased by 1.4 (Supplementary Table S1). Regarding inactivation, after 0.150 µg/µL exposition, the channel appeared to remain partially open at the more depolarized voltages, with relative conductance maintained around 10% from −25 mV onward (Figure 3c, right panel).

The inactivation kinetics were also analyzed for this channel. Figure 4b shows the effect of venom on fast and slow decay tau. Similar to Na_V_1.6, on Na_V_1.7, an almost 2-fold increase in the fast decay time constant was observed at 0.150 µg/µL (W = 36.0, *p* = 0.007). However, in Na_V_1.7, a small inhibition of inactivation was also observed in the slow component (t_(7)_ = 3.26, *p* = 0.0138) (Figure 4b, right panel). The presence of remaining current at the end of the pulse was also observed.

**Figure 5:**
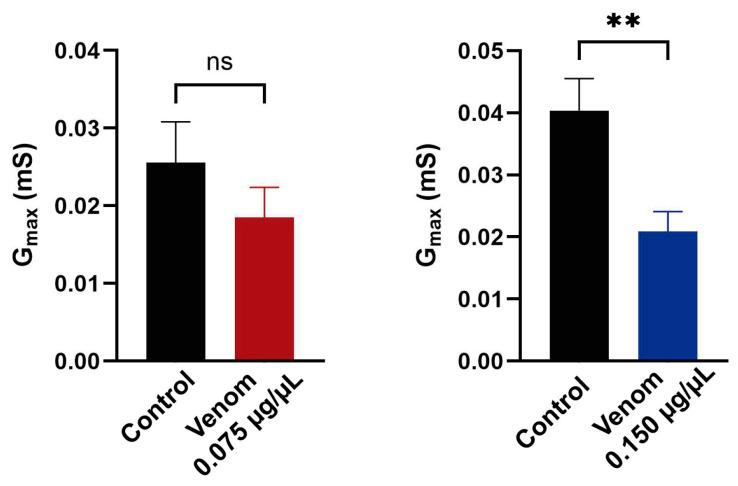
Na_V_1.7 maximum conductance (*G_max_*) was decreased by venom treatment. *G_max_* under control conditions (black) and under venom exposure at 0.075 µg/µL (red) or 0.150 µg/µL (blue) was calculated through the Boltzmann equation. Each value indicates mean ± s.e.m. The number of replicates (*n*) was 4 and 6 (for 0.075 and 0.150 µg/µL, respectively). ns = no significant, ** = *p* < 0.01.

For the Na_V_1.7 channel at 0.075 µg/µL, the reduction in the window current correlates with the smaller peak currents. However, at 0.150 µg/µL, the slight reduction in inactivation (Figure 3c, right panel), along with partial inhibition of inactivation (Figure 4b), does not correlate with the reduction in peak current observed in the I-V relationship (Figure 1c, right panel). To clarify this discrepancy, *G_max_* was calculated, revealing a statistically significant 50% decrease at 0.150 µg/µL compared to the control (t_(5)_ = 6.71, *p* = 0.001) (Figure 5).

In the invertebrate BgNa_V_1 channel, the activation curve remained mostly unaffected at both venom concentrations. However, at 0.150 µg/µL, the inactivation curve showed partial inactivation, inducing a larger window current compared to control conditions (Figure 3d).

Representative recordings of BgNa_V_1 (Figure 4c, left panel) showed that the venom also affected the inactivation kinetics, impacting both the fast and slow inactivation components. The fast inactivation time constant increased almost 4-fold after 0.075 µg/µL exposition (t_(7)_ = 4.02, *p* = 0.005) and increased approximately 3-fold after 0.150 µg/µL (W = 45.00, *p* = 0.0039) (Figure 4c, middle panel). The most pronounced effect, however, was observed in the slow inactivation component, where the decay tau increased more than 20-fold after 0.075 µg/µL exposition (t_(7)_ = 3.91, *p* = 0.005) and more than 19-fold after 0.150 µg/µL (t_(8)_ = 3.82, *p* = 0.005) (Figure 4c, right panel). Both increased window current and partial inhibition of inactivation can be related to the increase in the current observed in the I-V relationship.

### 3.3. Venom Effect on the Firing Frequency of Neurons Expressing Na_V_1.7-like Channels

Since *T. championi* venom showed an inhibitory effect on mammalian Na_V_1.7 at physiologically relevant voltages, such as −20 and −25 mV, and this channel has been associated with nociception, we decided to test the venom’s functional impact on firing frequency in invertebrate neurons that express homologous Na_V_1.7-like channels.

**Figure 6:**
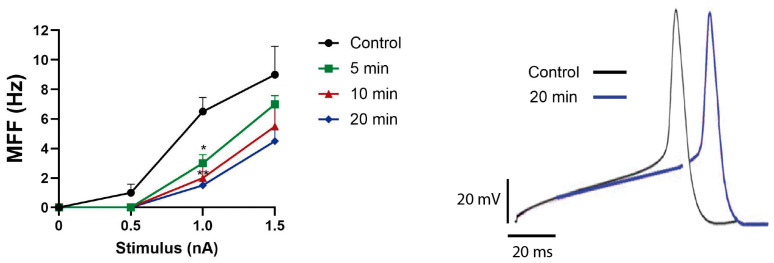
Firing frequency decreased upon exposure to *T. championi* venom. The left panel represents the Mean Firing Frequency (MFF) (Hz) recorded in *Helix* neurons. Stimulation protocol included three 500 ms stimuli of increasing intensity (0.5–1.5 nA) applied before (control, black circle) and after 5 min (green square), 10 min (red triangle), and 20 min (blue rhombus) of venom exposure (0.01 µg/µL). The right panel shows a representative recording of the first action potential evoked by a 1.5 nA stimulus before (black) and after 20 min of venom exposure (blue). Each value indicates mean ± s.e.m. (*n* = 4) * = *p* < 0.05, ** = *p* < 0.01.

Exposing the neurons to the venom at a concentration of 0.01 µg/µL completely abolished the mean firing frequency (MFF) induced by the smallest stimulus tested (0.5 nA) and decreased the MFF induced by 1.0 and 1.5 nA stimuli in a time-dependent manner (Figure 6, left panel), associated with slower depolarizations at the beginning of the action potential, delaying the reaching of the threshold (Figure 6, right panel). These results confirmed the dominant inhibitory effect of this venom on Na_V_1.7 channels.

### 3.4. Characterization of Dominant NaTxs Present in the Venom

To achieve a deeper understanding of the venom components responsible for the effects observed in *Helix* neurons expressing Na_V_1.7-like channels, we studied the effects of three of the most abundant toxins present in this venom. Given that only a small amount of toxins can be isolated in pure form from the venom, these data are shown for *n* = 2, allowing a general description of the effects.

#### 3.4.1. Tch3

**Figure 7:**
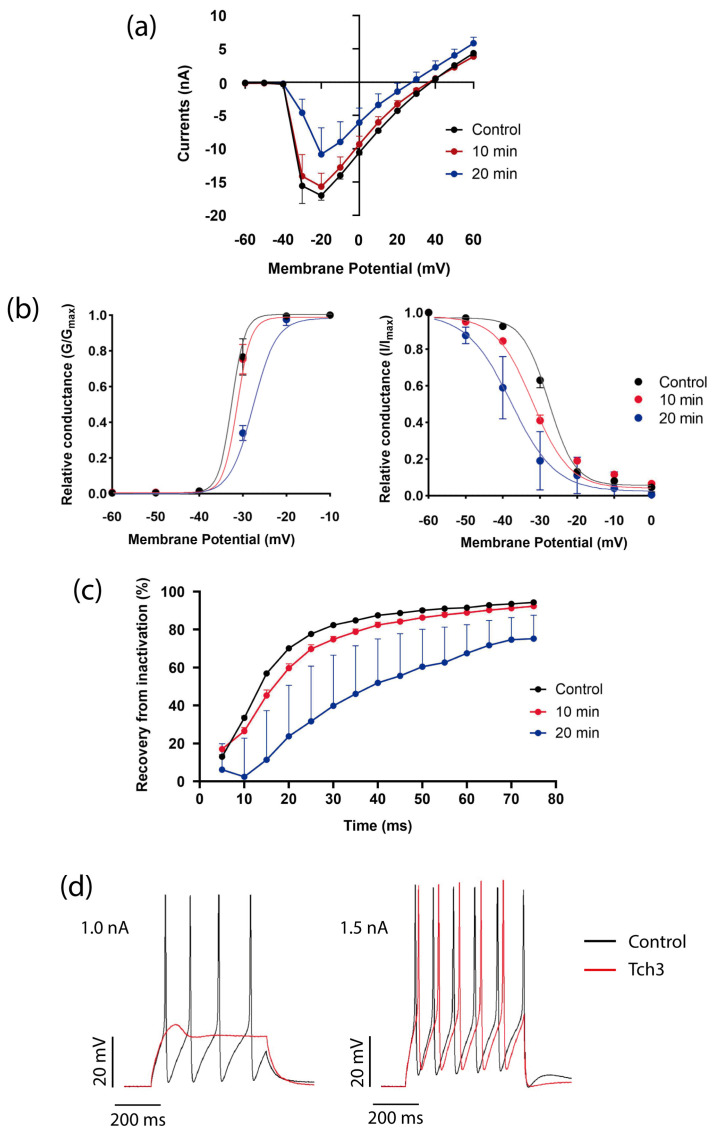
Tch3 decreased *Helix* Na_V_1.7-like currents by closing window current and decreased the recovery from inactivation and action potential firing. For I-V relationship (**a**), open probability of the activation and inactivation gates (calculated as relative conductance *G*/*G_max_* and *I*/*I_max_*, respectively) (**b**), and recovery from inactivation (evaluated after recovery times from 5 to 80 ms) (**c**); the effects were analyzed before (Control, black) and after 10 min (red), and 20 min (blue) of Tch3 exposure (0.01 µg/µL). Each value indicates mean ± s.e.m. (**d**) Representative recordings of action potential firing induced by 1.0 and 1.5 nA are presented before (Control, black) and after 20 min of 0.01 µg/µL Tch3 exposure (red).

One of the predominant toxins in *T. championi* venom is Tch3, a toxin with an 88% of homology with *T. obscurus* To7 [30]. The venom’s inhibitory effect was consistent with the toxin’s impact. Tch3 decreased *Helix* Na_V_1.7-like currents, especially after 20 min of exposure, compared to control currents (Figure 7a).

Consistent with this, we observed a positive shift of 3.94 mV in the activation curve (Figure 7b, left panel), reducing both *V*_1/2_ and voltage sensitivity (Supplementary Table S3). In addition, when the steady-state inactivation was evaluated, we observed a strong negative shift in the inactivation curve of −19.36 mV (Figure 7b, right panel) (Supplementary Table S4). The shift in both processes closed the window current and decreased its area by more than half (from 9.4 to 4.3 after 20 min) (Supplementary Figure S1).

Tch3 also affected the transition from the inactive to the closed state of Na_V_1.7-like channels, resulting in incomplete recovery from inactivation after 20 min of toxin exposure (Figure 7c).

Since this toxin showed an effect similar to the whole venom, we applied it to a single cell to test Tch3’s effect on neuronal AP firing. With a 1 nA stimulus, it was possible to see a complete blockage of the APs, and with a 1.5 nA stimulus, the frequency decreased, increasing interspike intervals (Figure 7d).

#### 3.4.2. Tch2

**Figure 8:**
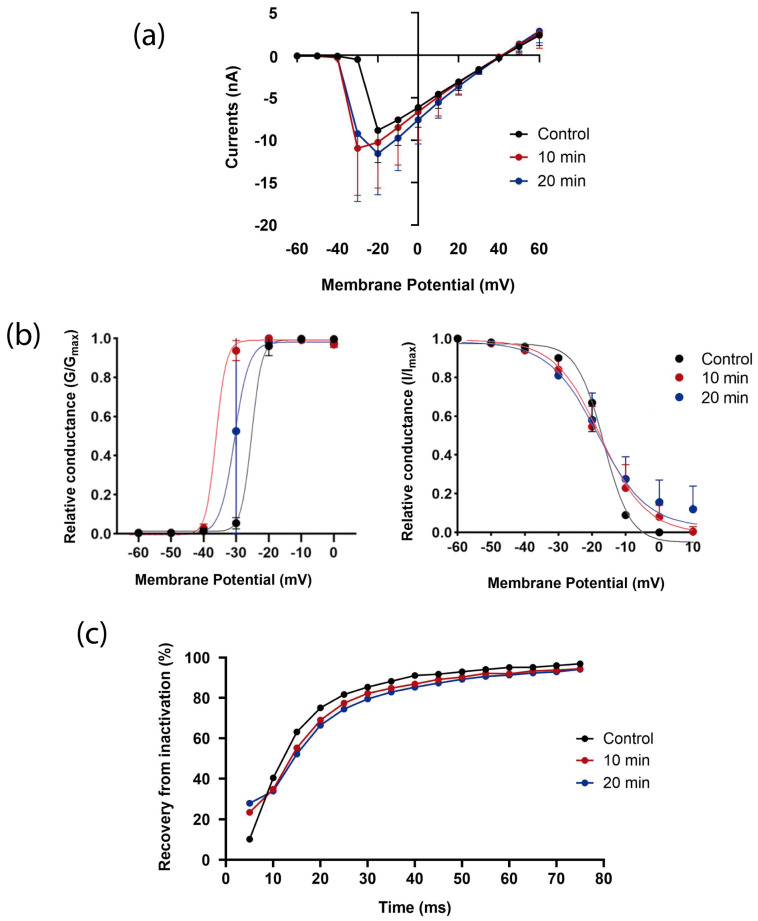
Tch2 induced the opening of the *Helix* Na_V_1.7-like channels at more negative voltages, increased window current, and decreased the recovery from inactivation. The effects were analyzed before (Control, black) and after 10 min (red), 20 min (blue), and 30 min (green) of Tch2 exposure (0.01 µg/µL): (**a**) I-V relationship. (**b**) The open probabilities of the activation and inactivation gates were calculated as relative conductances (*G*/*G_max_* and *I*/*I_max_*, respectively). (**c**) The recovery from inactivation was evaluated after recovery times from 5 to 80 ms and reported as the percentage of the first current. Each value indicates mean ± s.e.m.

Tch2 toxin was also analyzed. This toxin shares 91% homology with the To6 toxin [30]. Interestingly, Tch2 strongly increased the fast transient *Helix* Na_V_1.7-like currents evoked at −30 mV (Figure 8a). When the activation curves were analyzed, a negative shift was confirmed, reaching a shift of −9.20 mV after 10 min of exposition (Figure 8b, left panel) (Supplementary Table S3). Interestingly, this effect reversed after 20.

In terms of the impact of Tch2 on channel inactivation, almost no changes were observed in the *V*_1/2_ (Figure 8b, right panel). However, a decrease in voltage sensitivity was detected from 4.61 to 6.65 during the first 10 min of exposition (Supplementary Table S4). Taken together, the negative shift in activation and the decreased voltage sensitivity in inactivation lead to an increase in the window current, especially after 10 min of Tch2 exposure from 9.20 to 17.40 (Supplementary Figure S1).

Interestingly, the channel’s recovery from inactivation was increased within the first 5 ms after 10 min of exposure to the toxin (Figure 8c). However, a subsequent change in this pattern was observed, as the recovery percentage decreased slightly between 15 and 40 ms of interpulse interval.

#### 3.4.3. Tch4

**Figure 9:**
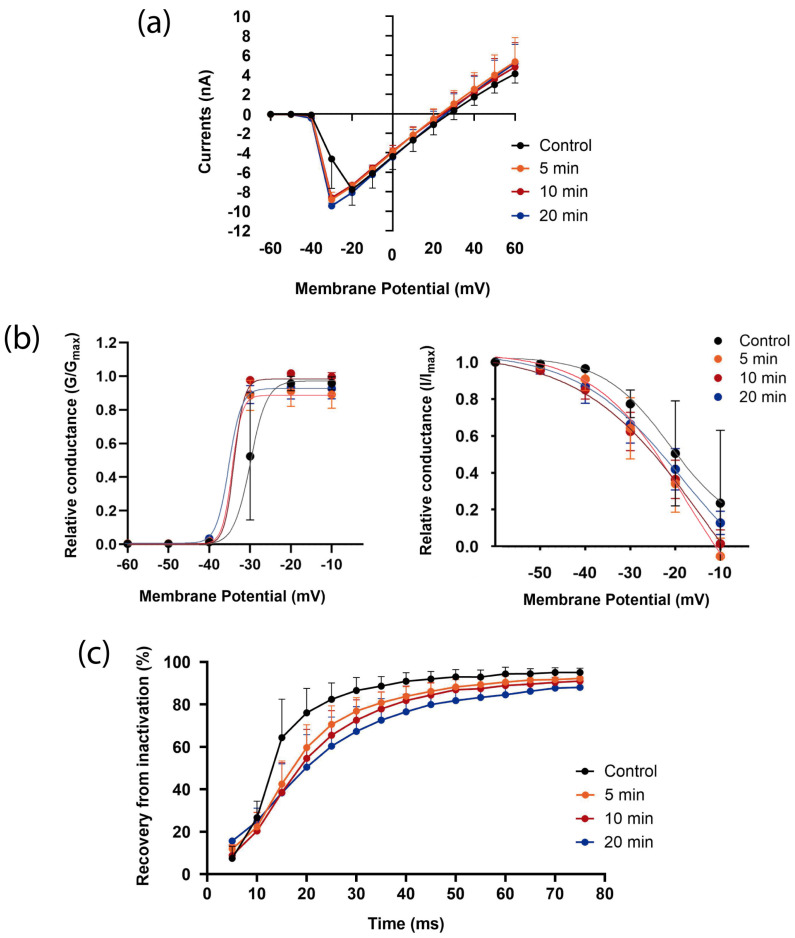
Tch4 induced the opening of the *Helix* Na_V_1.7-like channels at more negative voltages and decreased the recovery from inactivation. The effects were analyzed before (Control, black) and after 5 min (orange), 10 min (red), and 20 min (blue) of Tch4 exposure (0.01 µg/µL): (**a**) I-V relationship. (**b**) The open probabilities of the activation and inactivation gates were calculated as relative conductances (*G*/*G_max_* and *I*/*I_max_*, respectively). (**c**) The recovery from inactivation was evaluated after recovery times from 5 to 80 ms and reported as the percentage of the first current. Each value indicates mean ± s.e.m.

Tch4 toxin of *T. championi* shares high homology with To13 of *T. obscurus* [30]. As with Tch2, Tch4 increased *Helix* Na_V_1.7-like currents evoked at −30 mV (Figure 9a). This effect was evident from 5 min of toxin exposure and remained stable throughout the recording period. This finding agrees with a negative shift of 4.73 mV in the activation curve (Figure 9b, left panel), together with a bigger voltage sensitivity (Supplementary Table S3).

Analyzing the impact of Tch4 on channel inactivation, a negative shift of 6.31 mV in the steady-state inactivation (Figure 9b, right panel) was observed, together with a 3 times smaller voltage sensitivity (Supplementary Table S4). No substantial changes were observed in window currents (Supplementary Figure S1).

In terms of its effects on recovery from inactivation, Tch4 primarily caused a slower recovery through almost all the interpulse intervals. This was evident after 5 min of exposure and remained up to 20 min (Figure 9c).

### 3.5. Structure and Effect of Tch3 in Mammalian Systems

#### 3.5.1. Effect of Isolated Tch3 on Murine Na_V_1.7

**Figure 10:**
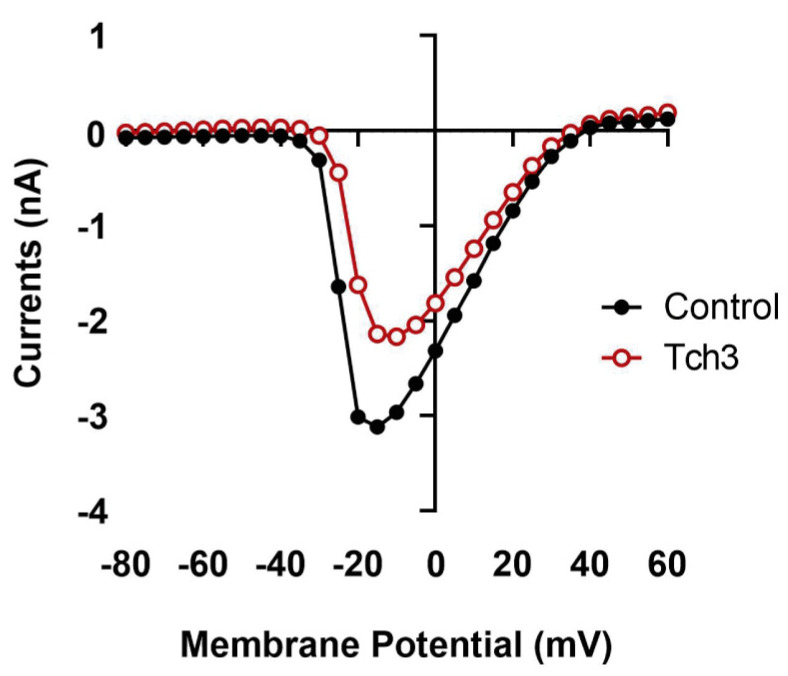
Tch3 decreased murine Na_V_1.7 currents. The currents were measured before and after Tch3 exposure (0.006 µg/µL).

Since Na_V_1.7 has been associated with the initiation of nociceptive transmission, it was interesting to evaluate in depth a toxin that inhibits its currents. Due to the challenging isolation process and the limited availability of this toxin, only one cell was used to assess its effect on oocytes expressing the murine Na_V_1.7 channel. Interestingly, as expected, Tch3 decreased Na^+^ currents (Figure 10), confirming the results observed in *Helix* neurons.

#### 3.5.2. Structure of Tch3 and Its Interaction with Human Na_V_1.7

The three-dimensional structure of the 57-amino acid toxin (sequence: EALDGYPLSKNNYCKIYCPNDEVCKDTCKHRAGATNGKGDCIWQTCYCYDVAPGTK) was predicted using AlphaFold ColabFold, yielding high-confidence structural models with excellent validation metrics. Sequence coverage analysis revealed a robust alignment depth throughout the protein, with particularly strong coverage in the central core region (positions 15–45), indicating reliable evolutionary information for structural prediction. Predicted Aligned Error (PAE) matrices for the five generated models showed predominantly low error values (blue regions) with minimal high-error zones, demonstrating consistent and accurate relative positioning of structural elements. Most importantly, per-residue confidence scores (pLDDT—predicted Local Distance Difference Test) indicated excellent predictive quality, with the functionally critical central region achieving pLDDT values above 80 and several segments exceeding 90—representing very high confidence by AlphaFold standards. The N- and C-terminal regions displayed lower confidence scores, typical for flexible terminal domains in small, disulfide-rich toxins (Supplementary Figure S2). Collectively, these validation metrics demonstrate that the predicted structure provides a reliable foundation for functional analyses and drug design applications, with the central region, which contains the putative active site, showing particularly high structural confidence.

**Figure 11:**
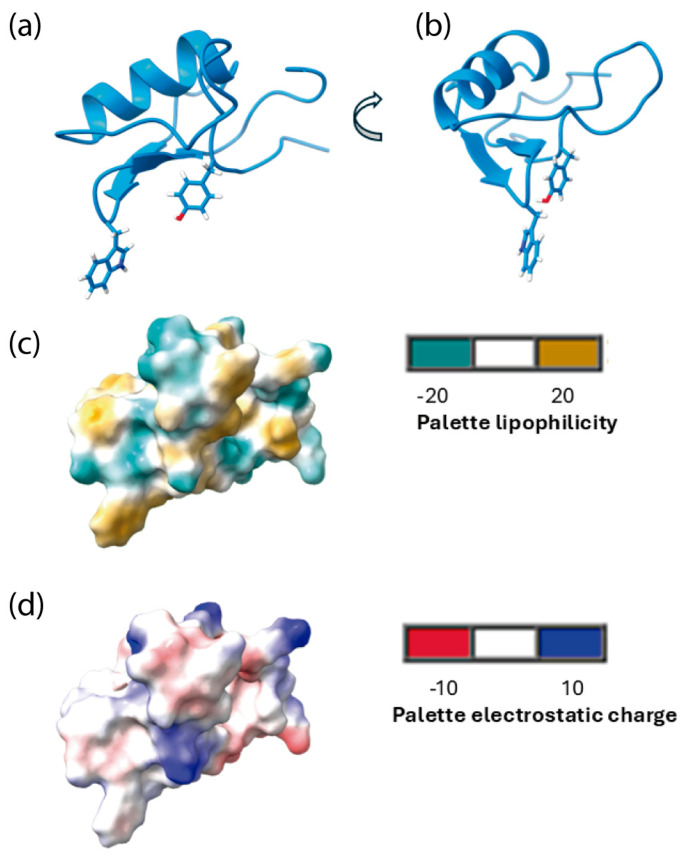
Structural model of the scorpion toxin Tch3: (**a**) Ribbon representation of Tch3. (**b**) Ribbon representation rotated along the vertical axis. (**c**) Molecular surface colored according to lipophilicity from hydrophilic/polar (teal/blue) regions to lipophilic/hydrophobic (yellow/orange) regions (color scale shown on the right panel). (**d**) Molecular surface colored according to electrostatic potential from negative potential (red) regions to positive potential (blue) regions (color scale shown on the right panel). Both color scales are arbitrary, and white represents neutral regions.

The analysis showed that Tch3 adopted an αββ conformation (Figure 11a,b). In addition, Figure 11c,d also show the prediction of surface lipophilicity and electrostatic charges.

**Figure 12:**
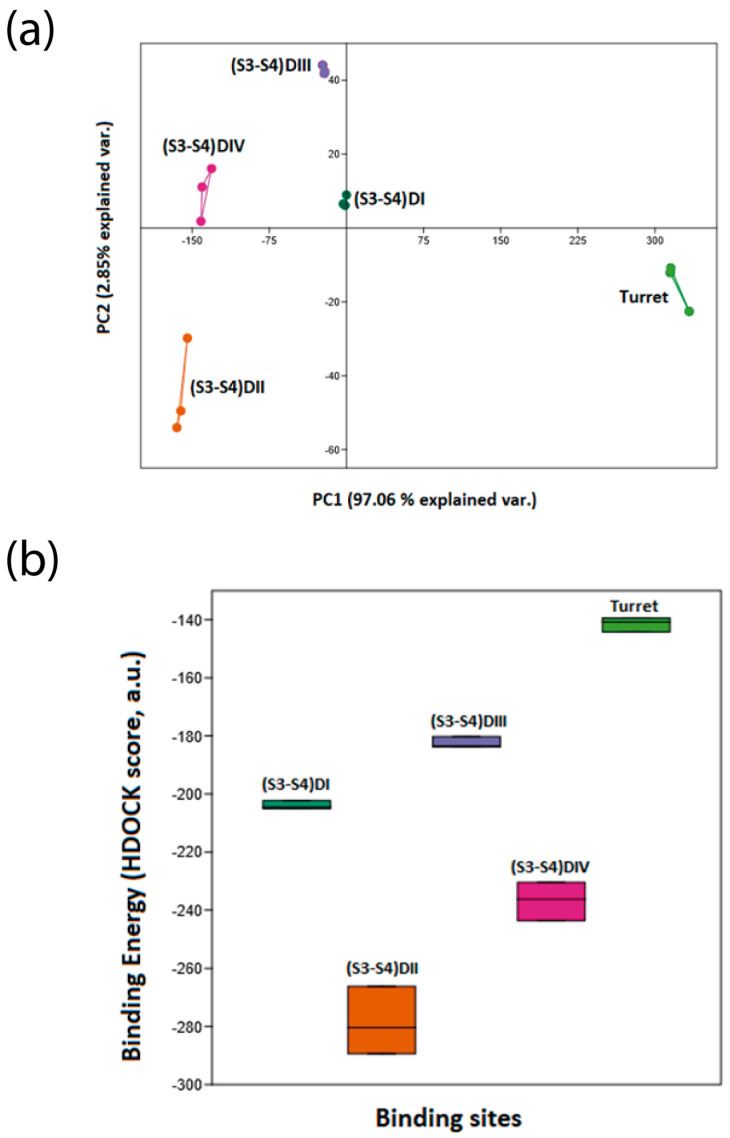
Tch3 toxin showed higher affinity for the S3–S4 loop in human Na_V_1.7 domain II: (**a**) Principal Component Analysis (PCA) of HDOCK binding energy scores for the five proposed binding sites of the Na_V_1.7 channel interacting with the Tch3 toxin showed a higher affinity for channel domain II. Each cluster (triangles) represents the dispersion of the most negative (highest affinity) docking energies obtained from three replicates. The first principal component (PC1), which explains 97.06% of the total variance, distinguishes sites by their relative binding affinity. Clusters located toward the lower-left quadrant correspond to higher-affinity interactions, whereas those toward the upper-right indicate lower affinity. The (S3–S4) loop of domain II (DII) exhibits the strongest predicted binding, followed by the (S3–S4) loop of domain IV (DIV), while the (S3–S4) loops of domains I (DI) and III (DIII) and the turret region display progressively weaker affinities. (**b**) Boxplot showing the distribution of binding energies (HDOCK, arbitrary units) for the five evaluated interaction sites (DI, DII, DIII, DIV, and Turret). Each box represents the median and interquartile range of three independent replicates. The upper and lower limits for each box (whiskers) denote the minimum and maximum values.

To identify the receptor site of the toxin, in the principal component (variance–covariance) plot (Figure 12a), the dispersion of data points was calculated from the most negative binding energy values (highest affinity) obtained from the three docking replicates for each of the five proposed binding sites of the Na_V_1.7 channel interacting with the Tch3 toxin. The plot shows that each binding site forms tightly grouped clusters, indicating good reproducibility across replicates.

A dispersion pattern toward the lower-left quadrant of the plot corresponds to more negative binding energies, reflecting higher binding affinity. In contrast, dispersions toward the upper-right quadrant indicate weaker affinities or resistance to binding. The first principal component (PC1) accounts for 97.06% of the total variance, representing the main axis of affinity variation, while the second principal component (PC2) explains only 2.85%.

Among the analyzed sites, the (S3–S4) loop of domain II (DII) exhibits the strongest predicted affinity, followed by the (S3–S4) loop of domain IV (DIV). In contrast, the (S3–S4) loops of domains I (DI) and III (DIII) display weaker binding effects, and the turret region shows the lowest affinity overall.

Visually, the boxplot in Figure 12b showed clear differences in the binding energies (HDOCK, arbitrary units) among all evaluated binding sites. Each treatment exhibited distinct energy distributions, consistent with the statistical analysis. The interaction corresponding to treatment D2, which represents the toxin binding to the S3–S4 loop of DII, displayed the most negative mean energy value, indicating the highest binding affinity among all tested sites. These visual differences reinforce the statistical finding that all treatments differ significantly from one another.

**Figure 13:**
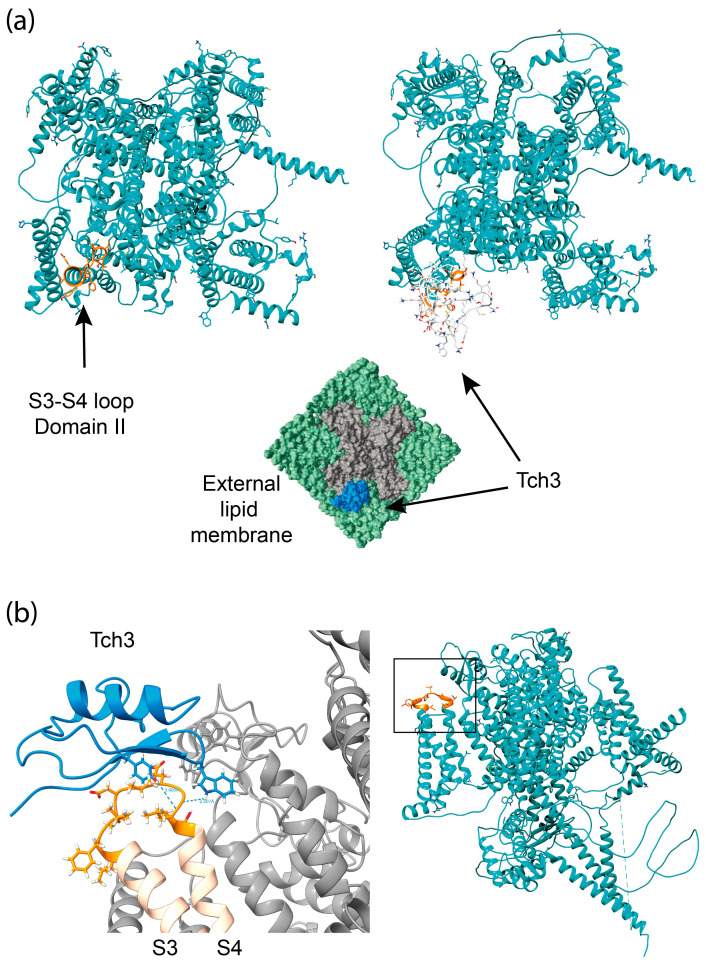
Tch3 Interaction with human Na_V_1.7: (**a**) Shows the hNa_V_1.7 (cyan structure) from an upper view, S3–S4 loop of the voltage sensor domain II (left panel, orange) acts as the receptor site for the Tch3 toxin (right panel, white), lower panel represents the Tch3 toxin (blue) binding to the channel (gray) in the presence of the membrane (green). (**b**) The right panel shows the side view of the hNa_V_1.7 (cyan) with the S3–S4 loop depicted in orange, the site market was zoomed in the left panel, showing the channel (gray), and 0the αββ structure of Tch3 toxin (blue) interacting with the S3–S4 loop of domain II (yellow and orange). The principal hydrogen bonds involved in the interaction presented hydrogen–acceptor distances of 3.900 Å and 2.607 Å (light blue dotted lines in left panel).

Figure 13a shows Na_V_1.7 from an upper view, depicting the S3–S4 loop of domain II in orange in the left panel, and with the Tch3 bind in the right and lower panels. Two hydrogen bond interactions were identified between the *T. championi* toxin Tch3 and the S3–S4 loop of domain IV in the Na_V_1.7 channel model (Figure 13b). The first hydrogen bond involves the backbone amide group of Leu874 from Na_V_1.7, acting as the donor, and the hydroxyl group of Tyr6 from Tch3, acting as the acceptor, with a donor–acceptor distance of 4.806 Å and a hydrogen–acceptor distance of 3.900 Å. The second hydrogen bond occurs between the indole nitrogen (NE1) of Trp43 from Tch3, acting as the donor, and the backbone carbonyl oxygen of Gly873 from Na_V_1.7, acting as the acceptor, with a donor–acceptor distance of 3.436 Å and a hydrogen–acceptor distance of 2.607 Å. These interactions suggest that Tch3 establishes stable hydrogen bonding contacts with the S3–S4 loop region, potentially contributing to its high binding affinity for the Na_V_1.7 channel.

## 4. Discussion

In the present study, we used heterologous expression of multiple Na_V_ channels in *Xenopus laevis* oocytes to perform a screening of the effects of *T. championi* venom. We chose this model because *Xenopus* oocytes have been widely used to study the ion channel properties in a model cell, free from other endogenous channels and responses [39]. In addition, we used the invertebrate C1 neuron to analyze the functional effect of specific toxins and complete venom on currents and potentials. We chose this model because invertebrate neurons exhibit neuronal function similar to that of mammalian neurons [40], and given the high level of conservation of functional processes, such as APs and Na^+^ currents, it is possible to study mechanisms in this simpler system and extrapolate them to more complex ones [41]. In this context, the serotonergic neuron C1 from the cerebral ganglion of *Helix* snails is commonly used due to its easy identification and high resistance to manipulation in vitro, and a Na_V_1.7-like channel has been identified on its membrane [42,43].

From a biological perspective, the cockroach is one of the most common prey for scorpions. And former studies have shown that Panama’s *T. championi* toxins cause insect paralysis in crickets [29]. As a consequence, in the present study, we tested the cockroach Na^+^ channel BgNa_V_1, and an increase in currents was observed at the highest venom concentration tested (Figure 2), along with bigger window currents (Figure 3d) and inhibition of inactivation (Figure 4c). It has been previously reported that toxins such as AaH II (from *Androctonus australis*) and LqhαIT2 (from *Leiurus quinquestriatus hebraeus*) also inhibit the inactivation of BgNa_V_1 currents, leading to prolonged APs in isolated giant cockroach axons [44]. The latter induces flaccid paralysis in fly larvae [12,45,46]. Also, fractions of crude venom from the *Latrodectus geometricus* spider demonstrated inhibition of inactivation in insect Na_V_ channels [47].

It is possible to suggest that the increased currents and incomplete channel inhibition observed can induce longer depolarizations in neurons and muscles, since impairment of fast and slow inactivation can cause persistent Na^+^ currents. Eventually, these effects will cause a failure in AP generation and lead to flaccid paralysis [48]. Such paralysis could allow the scorpion to easily feed on the immobilized prey. Since venoms are complex mixtures of toxins, it is necessary to identify whether the effects on the BgNa_V_1 channel are caused by one or several toxins with high selectivity for insect targets, which could make it a potential candidate for insecticide development [49,50].

In addition, in the present study, we also showed the influence of *T. championi* venom on mammalian Na_V_ isoforms, and we showed a clear trend with increased currents carried by Na_V_1.2 (Figure 1a) and a significant increase in Na_V_1.6 (Figure 1d), in contrast to the decreased currents through Na_V_1.7 (Figure 1e).

Na_V_1.2 is predominantly expressed in mammals during early development, where it is the primary Na_V_ channel in axons; later, it is replaced by Na_V_1.6 [51]. In adults, both Na_V_1.2 and Na_V_1.6 are located in the axon initial segment (AIS), but they serve different roles in AP generation, with Na_V_1.2 having a higher activation threshold [52,53].

In the case of Na_V_1.2, we observed similar currents at more negative potentials and slightly increased peak currents at more depolarized potentials (Figure 1a). This was accompanied by small positive shifts in the voltage sensitivity of both activation and inactivation, slightly moving the window current to more depolarized potentials (Figure 3a), leaving voltage dependency mostly unchanged and slightly increasing *G_max_*. Even if none of these effects reached statistical significance, their combined effect can correlate with the trend observed in I-V relationships. In the literature, some scorpion toxins have shown similar effects. For example, the toxin MeuNaTxα-1 also increased Na_V_1.2 currents with a minimal effect on inactivation, similar to our venoms, which was linked to initiating nociceptor sensitization through an indirect downstream mechanism [54]. However, following the classic classification, toxins affecting voltage sensitivity (β toxins) would typically induce a shift towards more polarized potentials, such as Ts17 [20], TsVII, Css VI, Css IV, and Css II [55], in contrast to the small depolarizing shift we observed for *T. championi* venom.

Interestingly, MeuNaTx α-1 (from *Mesobuthus eupeus*) induces a threefold increase in Na_V_1.2 current and significantly impairs inactivation in Na_V_1.6 currents [54], which is consistent with our findings using *T. championi* venom (Figure 4). Inhibition of inactivation is a hallmark of α-toxins’ action, commonly seen in venoms from the Buthidae family. These toxins bind to receptor site 3 in the domain IV of the Na_V_ channel’s α-subunit, preventing the outward movement of the voltage sensor and thus blocking inactivation [14,56,57]. Other examples of such toxins include Lqh2, LqhaIT, Lqh3 [54], and Ts4 [18], all of which similarly inhibit inactivation and increase inactivation time constant, as with the venom tested in this work (Figure 4, right panels).

Na_V_1.6 is the most abundantly expressed Na_V_ channel in mammalian central and peripheral nervous systems, especially in regions such as the AIS and nodes of Ranvier [58]. It plays a crucial role in lowering the voltage threshold for AP generation [59] and is implicated in increased neuronal hyperexcitability during epileptogenesis [60].

Here, we observed increased peak currents (Figure 1d), likely due to slower inactivation, and a substantial positive shift of +26 mV in inactivation *V*_1/2_, ultimately leading to incomplete inactivation and persistent currents (Figure 3b and Figure 4a). The inhibition of inactivation induced in this channel showed higher sensitivity to *T. championi* venom, which may be related to structural differences in this channel’s isoform. It has been suggested that Na_V_1.6 (located in chromosome 15 in humans) evolved independently of the other Na_V_s (most of them located in chromosomes 2 and 9) [61]. As a separate branch of the family, Na_V_1.6 can exhibit properties different from those of the other Na_V_s, making it more sensitive to venom components. As an example of these differences, toxins such as the β-toxin Cn2 or derivatives such as the 4,9-anhydrotetrodotoxin have been shown to specifically bind and affect Na_V_1.6 at concentrations that have minimal effects on other Na_V_s [62].

Functionally, the inhibition of inactivation and the consequent increase in current could lead to prolonged depolarizations in axon terminals and elevated neurotransmitter release [63]. This suggests a theoretical epileptogenic potential in the venom at the higher concentrations tested. Moreover, evidence suggests that heightened activation of Na_V_1.6 can promote inflammation [64], a critical mechanism deployed by scorpion venom [63].

Interestingly, when the effect on Na_V_1.7 was analyzed, *T. championi* crude venom showed a dual effect on its currents. On one side, at positive potentials, venom increased currents (Figure 1e) and decreased inactivation (Figure 4b); this effect could prolong APs and increase neuronal excitability. Similar effects have been reported for toxins such as Ts2 and Ts5, which inhibit the rapid inactivation of Na_V_1.6 and Na_V_1.7 [19]. However, this effect was evident at positive potential, where the depolarizing phase is almost finished [65], decreasing its physiological impact on AP width. On the other hand, we also observed a reduction in Na_V_1.7 peak current at negative voltages (Figure 1e). These potentials are more relevant for AP firing, since AP threshold in dorsal root ganglion neurons expressing Na_V_1.7 has been reported around −20 mV [65]. These decreased currents at this level can be associated with a substantial reduction in the channel’s maximum conductance (Figure 5). In summary, this venom could impair neuronal excitability, thereby reducing AP firing.

Consistent with this, exposing *Helix* neurons expressing Na_V_1.7-like channels to *T. championi* venom supported the effect observed in *Xenopus* oocytes. It was possible to confirm a decreased firing rate, along with a slower depolarizing phase (Figure 6). The venom of the scorpion *Buthus tamalus* also showed the capacity to suppress AP firing in mollusk neurons at 0.1 µg/µL [66]. In addition, the venom from a sister species, *T. bahiensis*, at a concentration of 0.01 µg/µL, induced a blockade in AP generation and muscle twitches in mammalian neuromuscular preparations [67]. Some of these findings also agree with observations from studies on synaptic transmission inhibitors derived from tarantula toxins, which evoked reductions in AP slopes [68].

Analyzing the most abundant potential neurotoxins in *T. championi* venom, we identified that Tch3 is a toxin that exerts the inhibitory effect over Na_V_1.7 currents and AP firing. Identified by Díaz and colleagues (2023), its sequence was also found in *T. jaimei* (named Tja3) and *T. dedoslargos* (named Tde3), which shared 88% homology with To7 from *T. obscurus* [30]. Also, To7 shares 69 and 63% identity with the neurotoxins TdNa10 and TdNa9 from *T. discrepans* [69]. All these sequences display a resemblance to α-NaTx, with a core domain similar to Old World scorpion toxins [49,69], whose species induce the most significant harmful effects in children [70]. However, our functional analysis cannot categorize the Tch3 toxin as an α-NaTx, even though it shows sequence similarities with those from various scorpions from the same genus.

The decreased currents after Tch3 exposition correlated with smaller window currents (Figure 7a,b and Supplementary Figure S1). A diminished probability of channel opening, resulting from a positive shift in the activation curve and a reduction in voltage dependence, which requires a greater voltage change to transition between closed and open states. Together with an inactivation that occurred at more negative potentials, as evidenced by a negative shift and a less steep slope. Collectively, led to less than half of the window current, culminating in a reduction in macroscopic currents.

Regarding the time required to achieve a significant decrease in current, Tch3 induced a significant change in activation and inactivation after 20 min of exposure, indicating a non-immediate response. This observation aligns with what has been described for other *Tityus* NaTxs, such as TsIV-5, which have been reported to require 10–20 min to reach a steady-state effect [71]. Similarly, when the binding kinetics of the toxin Lqh III (from *Leiurus quinquestriatus hebraeus*) was analyzed in cardiac Na_V_s, it showed a time constant of association of 900 s (15 min) at a concentration as high as 5 nM. Interestingly, despite its low association rate, Lqh III showed the strongest effects on channel function among the toxins analyzed [72]. Additionally, an experimental factor that likely contributes to the delay in Tch3 effects, independent of toxin kinetics, is the diffusion rate of venom components across the extracellular medium volume (~2 mL), since the molecules are not applied directly to the cell to maintain patch integrity.

This latency in the toxin effects has also been identified in studies with prey insects, exposing crickets to Tma2 and Tma3 from *T. macrochirus* (classified as insect α-NaTx), where initial symptoms of disorientation and paralysis were observed, and insect death was recorded up to 4 h later [73]. This effect is non-lethal at the moment, but it is likely sufficient to facilitate predation on the animal.

Similar to Tch3, the Tc49b toxin, classified as α-NaTx, deviates from the conventional characteristics of this toxin type. Notably, it exerts an almost complete blocking effect on the current at a concentration of 100 nM in rat cerebellar granule cells [74], in agreement with our results. Inhibition of Na_V_1.7 has also been reported for the tarantula *Thrixopelma prurient* toxin ProTx-II. ProTx-II exhibits noteworthy specificity for human Na_V_1.7 channels. Notably, it induces a significant shift in activation toward more positive potentials, similar to the impact observed with Tch3 toxin. In addition, it substantially reduces channel conductance [68] as observed with *T. championi* venom.

These effects, together with the smaller recovery from inactivation (Figure 7c), evoked a decrease in AP firing in this neuron (Figure 7d). This remarkable effect is particularly significant, as it is known that Na_V_1.7 is primarily expressed in peripheral somatic neurons and plays a pivotal role in regulating sensory neuron excitability, for example, being a threshold channel for AP firing in nociceptors [65,75,76]. Moreover, this channel is upregulated, and its activation threshold decreases during inflammatory and neuropathic pain [77]. Besides, gain-of-function mutations in the *SCN9A* gene, which encodes Na_V_1.7, have been linked to neuropathic pain conditions, and loss-of-function mutations have been associated with congenital insensitivity to pain [78].

A behavior similar to Tch3 has been described for the local anesthetic QX-314, a derivative of lidocaine. QX-314 induces a shift in the activation *V*_1/2_ to more positive voltages, while simultaneously inhibiting peak current in Na_V_1.7 channels [79]. Through the analysis of mutant channels, Klasfauseweh and collaborators concluded that pore blocking and restriction of S4 voltage-sensor movement can result from multiple interactions between the positive charges of QX-314 and the channel’s negatively charged residues.

The convergence in mechanisms of action between analgesics and scorpion toxins opens several possibilities as potential agents targeting pathologies associated with pain and nociception. For example, the toxin BmK IT2 (from *Buthus martensi* Karsch) has shown analgesic potential derived from its capacity to inhibit Na^+^ currents [80,81]. Similarly, Suzetrigine (VX-548) is an analgesic developed after the study of molecules as NaTxs, used to treat moderate-to-severe acute pain due to its capacity to selectively inhibit Na_V_1.8 [82]. Interestingly, upon oral administration, suzetrigine reaches plasma concentrations of about 0.62 μg/mL [82], which corresponds to ~1.3 μM. In agreement, the concentrations tested of Tch3 in oocytes (0.006 μg/μL) and in *Helix* neurons (0.01 μg/μL), assuming a rough mean molecular weight of 6–7 kDa for NaTx, were about 1–2 µM.

As pointed out before, it has been reported that the α-NaTxs prevent rapid channel inactivation by binding to an extracellular loop between S3 and S4 segments of the Na_V_’s domain II, called receptor site 3 [83]. Due to their proximity to the S4 voltage sensor in domain IV (IVS4), these toxins keep the sensor in an internal, non-active position, delaying inactivation. On the other hand, the β-NaTxs promote channel opening by binding to the extracellular loop between S3 and S4 of domain II, called receptor site 4. During channel activation, the S4 voltage sensor of domain II is translocated extracellularly, where it interacts with the β-NaTxs, anchoring it in an external activated position [1,84].

When modeled, Tch3 toxin showed a structure composed of one α-helix and two antiparallel β-sheets (Figure 11). This kind of structure is common in several invertebrate NaTx, such as Sm2 from the centipede *Scolopendra morsitans*, discrepin from scorpion *T. discrepans*, agitoxin 2 and LqhαIT from the scorpion *Leiurus quinquestriatus hebraeus*, and Lqq3 from *Leiurus quinquestriatus quinquestriatus* [16]. In addition, even when shared sequence homology with α-NaTxs [30,69], its interaction with hNa_V_1.7 shows higher affinity at receptor site 4 (Figure 12), suggesting it is a β-NaTx. In this regard, β-NaTxs toxins have been subclassified as typical excitatory β toxins and depressant β toxins based on their in vivo effects on insects, inducing fast contraction paralysis or progressive flaccid paralysis [5,85]. Altogether, the functional and structural analysis of different Na_V_1.7 would allow us to classify Tch3 as a depressant β-NaTx.

It has been proposed that depressant β-NaTxs trap the S4 voltage sensor in an intermediate state, preventing the channel from fully transitioning to the open state and reducing channel currents [5], an effect similar to what we saw over mammal Na_V_1.7 (Figure 10) and Na_V_1.7-like channels (Figure 7). Also, for *Tityus zulianus*’ Tz1 toxin, it was reported that it slows down the voltage sensor movement [85]. And tarantula toxins such as CcoTx1, CcoTx2, CcoTx3 (from *Ceratogyrus cornuatus*), and PaurTx3 (from *Phrixotrichs auratus*) inhibit Na_V_ channels by shifting *V*_1/2_ to more positive voltages. Finally, toxin ProTx-II from *T. pruriens* tarantula and µO conotoxin MrVIA from *Conus marmoreus* inhibit Na_V_ currents by stabilizing the voltage sensor from domain II in the resting state position [85].

Specifically, Xu and collaborators (2019) proposed that ProTx-II acts as an electrostatic gating modifier. This mechanism involves the introduction of positive charges into the S3–S4 loop of the voltage sensor domain II (the receptor site 4). The toxin neutralizes acidic residues with basic side chains, impeding the movement of S4 [86]. Since Tch3 was able to interact with receptor site 4 in hNa_V_1.7 (Figure 13), it may present a similar mechanism of action.

It was interesting that the other two toxins described in this work (Tch2 and Tch4) behaved as traditional β-toxins (Figure 8 and Figure 9). In this matter, β-toxins can be associated with the induction of repetitive firing in response to short currents, as observed in the presence of venom rich in β-NaTx, such as *Centruroides suffusus* venom [87].

Both Tch2 and Tch4 induced a negative shift in voltage-sensitivity (Figure 8b and Figure 9b, Supplementary Tables S3 and S4). For instance, Tch2 was also found in the scorpions *T. jaimei* (named Tja2) and *T. desdoslargos* (named Tde2), and shares 91% homology with To6, an α-NaTx from *T. obscurus*. Tch4 was also found in the scorpions *T. jaimei* (named Tja4) and *T. desdoslargos* (named Tde4) and has near identical sequences to To13 from *T. obscurus*, a toxin that shares sequence similarities with α- and β-NaTx [30,69]. Despite their high homology with α-NaTxs, our study showed that Tch2 and Tch4 behave like β-NaTxs. In *T. obscurus,* this kind of effect was described for To4, classified as a β-NaTx, where there is a shift in *V*_1/2_ to more hyperpolarized values in all human Na_V_ isoforms [88].

A similar lack of congruence between sequence homology and functional effects has been reported previously. For example, toxin Ts17 shared sequence homology with Ts5 and Ts3 (both α-NaTx) from the same species. However, a detailed electrophysiological analysis unambiguously characterized it as a β-NaTx [20]. This divergence was attributed to the highly conserved three-dimensional structure of both scorpion toxin groups, characterized by α-helices and 3 to 4 antiparallel β-sheets, featuring cysteine residues critical for disulfide bridge formation [89]. Noteworthy insights arise from molecular dynamics simulations conducted by Chen and Chung [90], indicating that both α- and β-NaTx bind to receptor sites 3 and 4, respectively, in a consistent orientation within the binding pocket [90]. This underscores that distinctions in functional outcomes may be more closely associated with specific amino acid residues pivotal to interactions within the receptor site.

When compared with toxins such as AaHIT from *Androctonus australis* (β-NaTx selective for insects), CssII and CssIV from *C. suffusus* (β-NaTx selective for mammals), and TsVII from *T. serrulatus* (β-like selective for mammals and insects), all of these produce an increase in the inward currents at −40 mV [55]. Similarly, in our data, Tch2 and Tch4 toxins increased the Na^+^ current at −30 mV.

The mechanism by which classical β-NaTx interacts with Na_V_ channels is known as the voltage sensor-trapping model. In this model, the toxin anchors to the loops between segments S3 and S4 of domain II, keeping the channel in a pre-active state, and making it more sensitive to voltage depolarization [84]. This increase in the probability of channel opening at more negative voltages could be associated with an excitatory effect of the toxin, similar to the effects observed with toxins like AaHIT from *A. australis* and Bj-xtrlT from *Buthotus judaicus* [55]. In these cases, rapid contraction paralysis occurs as an immediate, reversible effect, leading to spastic paralysis due to generalized skeletal muscle activation. This activation results from an excitatory presynaptic action on motor nerves, leading to repetitive firing [91].

We observed that the window current increases but reverses its effect after 20 min of exposure to Tch2 (Supplementary Figure S1), exhibiting a fast and reversible effect; however, it does not return to control values at the times tested. This partial recovery behavior has also been reported for some α-NaTxs, such as Tc49b and Ts4 [18,74]. From an evolutionary perspective, it can be associated with the immediate time required for the effect to be sufficient to immobilize the scorpion prey with its pincers, and there might not be strong selection for toxins with excessively high binding affinity. The reversibility of the toxins is consistent with observations that venom from *T. obscurus* shows a systemic effect hours after injection and reverses after 3 h in over 50% of animals [92].

An interesting observation is that in our two β-NaTx (Tch2 and Tch4), the slope factor (*k*) describing the steady-state inactivation increased, indicating a decrease in voltage dependence, as evidenced by the less steep slope of the inactivation curve (Figure 8 and Figure 9 and Supplementary Table S4). Similarly, in the case of the Tst1 toxin, most Na_V_ isoforms tested exhibit an increase in *k* of inactivation [93].

However, *T. championi* venom exerted an inhibitory effect, suggesting that these β-NaTxs constitute a smaller functional component of the venom. On the other hand, these two toxins also induced smaller recovery from inactivation at most interpulse intervals, an effect that can dampen firing frequency during repetitive firing. Similar observations were made for Ts17, a β-NaTx that decreased recovery from inactivation [20]. This suggests that, like Ts17, Tch2 and Tch4 stabilize a population of channels in an inactive state, preventing their return to the closed state and subsequent opening during further depolarizations, thereby contributing to the venom’s overall inhibitory effect on firing frequency.

## 5. Conclusions

The identification of distinct toxic mechanisms across Na_V_ isoforms in mammals and insects highlights the venom’s evolutionary adaptation to different channel isoforms, reflecting the dual role of these animals as predators and prey of scorpions. In addition, these findings open new avenues for therapeutic and scientific applications targeting Na_V_ channel subtypes, such as Na_V_1.7, particularly in the context of neurological disorders and pain management.

It is interesting to note that while the venom increased current in channels such as Na_V_1.2 and Na_V_1.6, possibly contributing to excitatory and inflammatory responses, it mainly reduced Na_V_1.7 currents, probably affecting nociceptors. In addition, *Helix* results confirm the presence of β-NaTxs in this venom (such as Tch2, Tch3, and Tch4), and venom results in oocytes suggest the presence of unidentified α-NaTxs, which induce inhibition of inactivation in channels such as Na_V_1.6 and Na_V_1.7. This suggests a potential dual role for *T. championi* venom, with excitatory effects in some cells and inhibitory effects in others. These highlight the complex mechanisms by which scorpion venoms modulate neuronal excitability.

We demonstrated that *T. championi* venom inhibited cellular excitability in neurons expressing Na_V_1.7-like channels, at least partially through the inhibitory action of the Tch3 toxin. In addition, since the Tch2 and Tch4 toxins decreased recovery from inactivation, it is likely that they reduced excitability during repetitive firing by increasing the accumulation of inactive Na_V_s. Altogether, Na_V_1.7 inhibition and Tch3 interaction with human channels suggest its potential therapeutic value in pain treatment. Future studies using animal models should be conducted to confirm this and further explore its therapeutic potential.

## Data Availability

The data presented in this study are available in the manuscript’s figures and tables; statistical analysis is available at https://github.com/GalitAkerman/CR-ScorpionVenom-Nav.git (accessed on 4 April 2026). Further information is available upon request.

## Supplementary Materials

The following supporting information can be downloaded at: https://www.mdpi.com/article/10.3390/biom16040552/s1, Figure S1: The window current changes depending on the toxin exposure; Figure S2: Tch3 structure prediction; Table S1: Biophysical parameters describing the open probability of the m particle of Na_V_s expressed in *Xenopus* oocytes; Table S2: Biophysical parameters describing the steady-state inactivation of Na_V_s expressed in *Xenopus* oocytes; Table S3: Biophysical parameters describing the open probability of the m particle of Na_V_1.7 from *Helix* neurons; Table S4: Biophysical parameters describing the steady-state inactivation of Na_V_1.7 from *Helix* neurons.
