# Supplementary material for: Electrophysiological Characterization of the Venom and Toxins from the Scorpion Tityus championi Targeting Voltage-Gated Sodium Channels and Molecular Modeling of Tch3, a Toxin with Therapeutic Potential for Pain Relief
Source: Biomolecules. 2026 Apr 8;16(4):552. doi: 10.3390/biom16040552 (PMC13113398; doi:10.3390/biom16040552)
Supplement: Supplementary file 1 [file biomolecules-16-00552-s001.zip › supplementary figures-biomolecules-4211669.pdf]

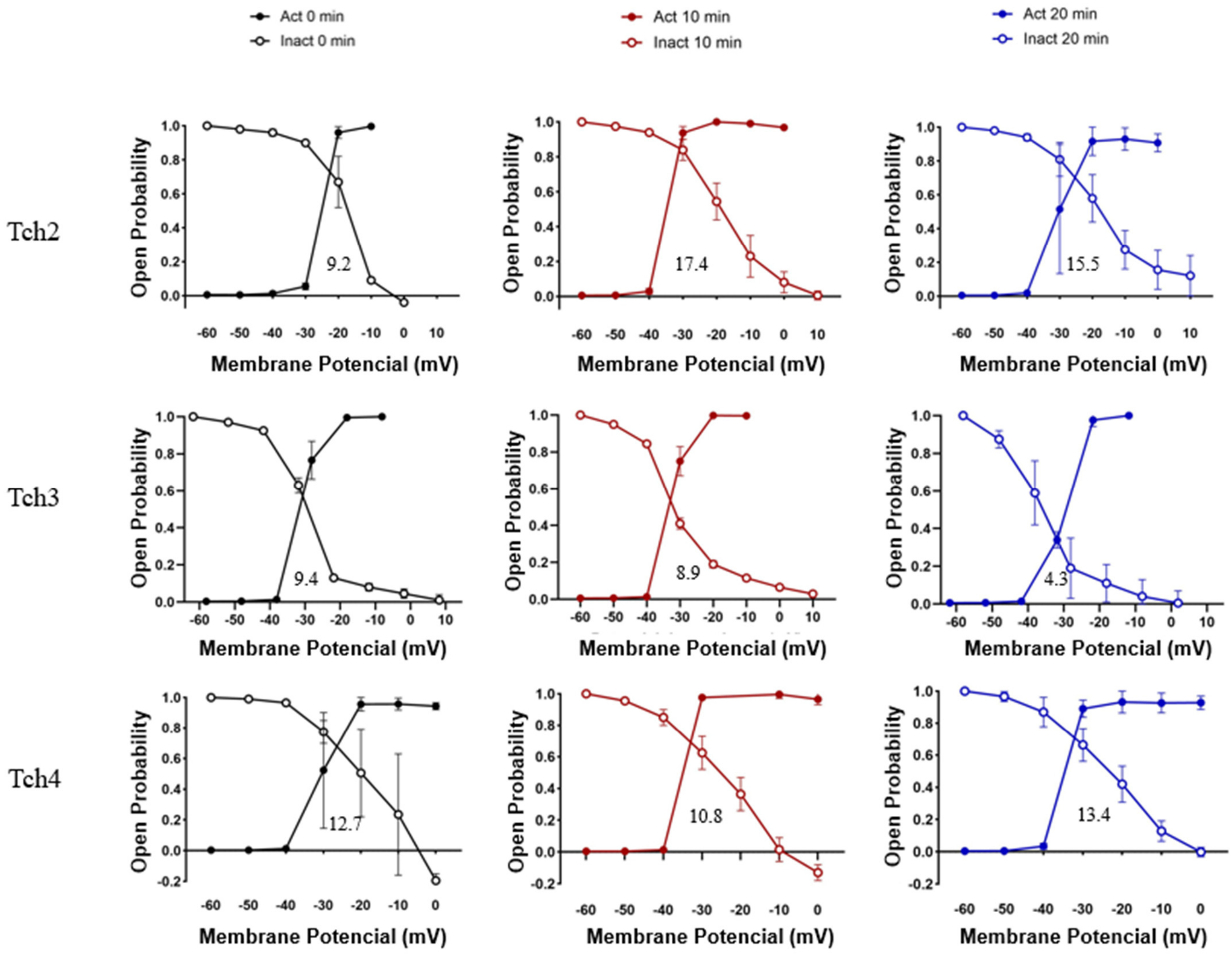

Figure S1. The window current changes depending on the toxin exposure. The window current (WC) is observed by combining the activation curves (closed circles) generated as relative conductance (G/G_{max}), and inactivation curves (open circles) calculated as relative current (I/I_{max}) in the same graph. The value of the window current area is denoted in each graph. The upper panel shows Tch2, the middle panel shows Tch3, and the lower panel shows Tch4. The control WC (black) was calculated before toxin exposure, and the effect of the toxins was calculated after 10 min (red), and 20 min (blue) of exposure (n=2). Each value indicates mean ± s.e.m.

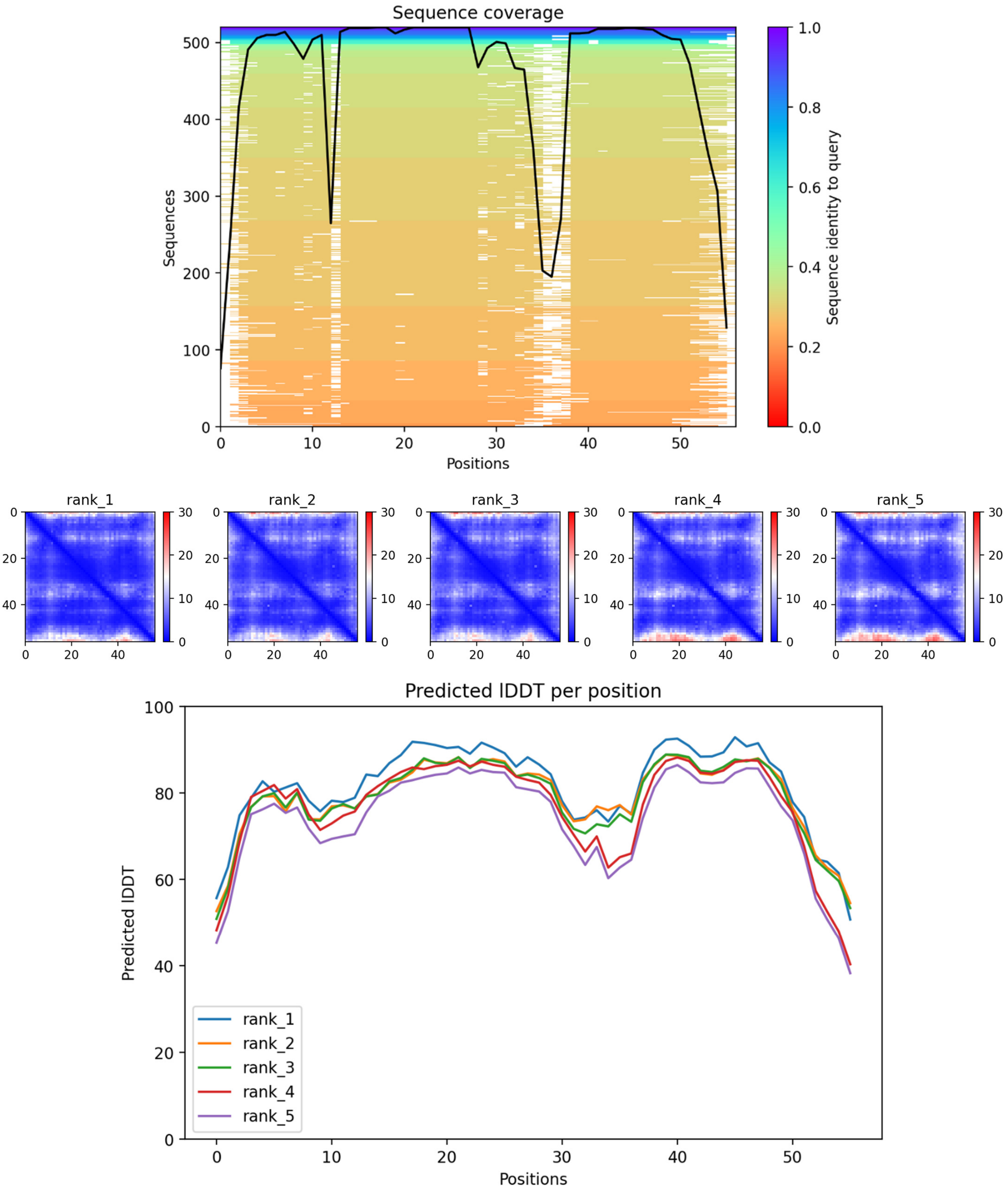

Figure S2. Tch3 structure prediction. The sequence coverage analysis (upper panel) showed strong coverage, especially in the central core region. Five models were generated (middle panel), and all of the Predicted Aligned Error matrices showed low error values (blue regions). In addition, the per-residue confidence scores (pLDDT - predicted local Distance Difference Test, lower panel) showed structural prediction reliability over 80 and 90 for most of the protein.
