# Supplementary material for: Electrophysiological Characterization of the Venom and Toxins from the Scorpion Tityus championi Targeting Voltage-Gated Sodium Channels and Molecular Modeling of Tch3, a Toxin with Therapeutic Potential for Pain Relief
Source: Biomolecules. 2026 Apr 8;16(4):552. doi: 10.3390/biom16040552 (PMC13113398; doi:10.3390/biom16040552)
Supplement: Supplementary file 1 [file biomolecules-16-00552-s001.zip › supplementary tables-biomolecules-4211669.pdf]

Table S1. Biophysical parameters describing the open probability of the m particle of Navs expressed in *Xenopus* oocytes. Values are derived from the fitting of the mean activation curve using the Boltzmann equation of channels exposed to different concentrations of *T. championi* venom. $V_{1/2}$ is the voltage corresponding to half-maximal activation in mV; *k* is the slope factor of activation.

Channel	0.075 µg/µL					0.150 µg/µL				
	Control		Venom		n	Control		Venom		*n*
	$V_{1/2}$	*k*	$V_{1/2}$	*k*		$V_{1/2}$	*k*	$V_{1/2}$	*k*	
**Nav1.2**	−29.67	4.25	−25.00	4.41	2	−36.18	3.22	−32.33	3.47	3
**Nav1.6**	−22.50	7.80	−12.00	7.18	2	−27.41	4.21	−25.84	4.91	3
**Nav1.7**	−21.43	3.59	−15.44	5.02	4	−25.07	2.94	−23.80	3.82	6
**BgNav1**	−47.00	2.93	−46.00	3.24	8	−43.87	4.21	−42.50	5.34	8

Table S2. Biophysical parameters describing the steady-state inactivation of Navs expressed in *Xenopus* oocytes. Values are derived from the mean inactivation curve using the Boltzmann equation from channels exposed to different concentrations of *T. championi* venom. $V_{1/2}$ is the voltage corresponding to half-maximal inactivation in mV; *k* is the slope factor of inactivation.

Channel	0.075 µg/µL					0.150 µg/µL				
	Control		Venom		n	Control		Venom		*n*
	$V_{1/2}$	*k*	$V_{1/2}$	*k*		$V_{1/2}$	*k*	$V_{1/2}$	*k*	
**Nav1.2**	−46.10	6.00	−42.19	7.35	2	−48.38	8.09	−42.28	7.10	3
**Nav1.6**	−48.10	7.35	−47.80	7.62	1	−53.75	6.89	−27.50	6.54	3
**Nav1.7**	−43.50	10.61	−43.00	10.93	4	−47.53	11.06	−47.50	12.12	6
**BgNav1**	−47.00	4.48	−46.50	4.94	8	−44.47	4.44	−44.70	5.28	8

Table S3. Biophysical parameters describing the open probability of the m particle of Nav1.7 from *Helix* neurons. Values are derived from the fitting of the mean activation curve using the Boltzmann equation of channels exposed to three toxins of *T. championi* venom. $V_{1/2}$ is the voltage corresponding to half-maximal activation in mV; *k* is the slope factor of activation. All data are shown from *n*=2, as mean ± s.e.m.

Toxin	$V_{1/2}$				*k*			
	Time (min)				Time (min)			
	0	5	10	20	0	5	10	20
**Tch2**	−25.55 ± 0.52	−30.08 ± 3.09	−34.75 ± 1.58	−29.69 ± 3.64	1.5 ± 0.35	1.40 ± 0.09	1.29 ± 0.16	1.85 ± 0.32
**Tch3**	−32.08 ± 0.77	−31.85 ± 0.71	−28.07 ± 0.46	−28.14 ± 1.61	1.72 ± 0.21	1.62 ± 0.005	2.66 ± 0.07	3.18 ± 0.41
**Tch4**	−29.57 ± 4.84	−34.72 ± 0.14	−34.02 ± 0.57	−34.30 ± 0.36	2.03 ±0.65	1.27 ±0.02	1.23 ± 0.03	1.63 ± 0.23

Table S4. Biophysical parameters describing the steady-state inactivation of Nav1.7 from *Helix* neurons. Values are derived from the mean inactivation curve using the Boltzmann equation from channels exposed to three toxins of *T. championi* venom. $V_{1/2}$ is the voltage corresponding to half-maximal inactivation in mV; *k* is the slope factor of inactivation. All data are shown from *n*=2, as mean ± s.e.m.

Toxin	$V_{1/2}$				*k*			
	Time (min)				Time (min)			
	0	5	10	20	0	5	10	20
**Tch2**	−16.22 ± 2.01	−16.77 ± 2.88	−17.82 ± 3.14	−18.79 ± 2.87	4.38 ± 1.25	4.72 ± 1.81	7.22 ± 0.43	7.89 ± 0.94
**Tch3**	−27.54 ± 1.08	−32.00 ± 0.53	−38.00 ± 3.77	−46.90 ± 2.23	4.61 ± 0.20	6.24 ± 0.30	6.65 ± 1.71	6.85 ± 1.72
**Tch4**	−16.33 ± 6.63	−26.24 ± 5.46	−22.33 ± 2.90	−22.64 ± 4.16	3.06 ± 2.73	5.19 ± 0.08	9.31 ± 1.44	10.55 ± 1.74
